# Comparison of the efficacy of different orthotopic neobladder reconstruction techniques following radical cystectomy for bladder cancer: a network meta-analysis

**DOI:** 10.3389/fonc.2026.1857787

**Published:** 2026-06-10

**Authors:** Zhengze Xi, Huaixi Ge, Jianhang Zhu, Qingbo Yang, Fei Xie, Xiaocheng Ma, Dongxu Zhang, Haitao Niu, Yonghua Wang

**Affiliations:** 1 Department of Urology, The Affiliated Hospital of Qingdao University, Qingdao, China; 2 Urinary Diseases Clinical Medical Research Center of Qingdao, Qingdao, China

**Keywords:** bladder cancer, functional outcomes, network meta-analysis, orthotopic neobladder, radical cystectomy

## Abstract

**Background:**

Orthotopic neobladder (ONB) reconstruction is increasingly utilized following radical cystectomy (RC) for bladder cancer (BC). However, the comparative functional performance of different ONB techniques remains inconclusive.

**Materials and methods:**

A systematic review and network meta-analysis were conducted in accordance with the PRISMA-NMA guidelines. The study protocol was prospectively registered in PROSPERO (CRD420251143294). PubMed, EMBASE, and the Cochrane Library were systematically searched from database inception to October 2025. Eligible studies included those enrolled patients undergoing RC with any ONB technique and reported at least one predefined outcome: maximum neobladder capacity (MNC), maximal flow rate (MFR), postvoid residual volume (PVR), daytime continence (DC), or nighttime continence (NC). A Bayesian random-effects model was applied to synthesize direct and indirect evidence and to rank interventions using the surface under the cumulative ranking curve (SUCRA).

**Results:**

Fifteen studies involving 1,129 patients and 14 ONB reconstruction techniques were included. Most techniques showed no statistically significant differences compared with the Studer pouch across core functional outcomes. However, the Studer pouch demonstrated superior DC compared with the Camey I pouch, while the ileocecal pouch achieved significantly lower PVR than the Studer pouch (both P < 0.05). SUCRA-based rankings indicated that the T pouch, Xing pouch, Ileocecal pouch, Y pouch, and Mainz pouch performed best for MNC, MFR, PVR, DC, and NC, respectively.

**Conclusion:**

Across five core functional outcomes, most ONB reconstruction techniques demonstrated comparable performance. SUCRA-derived rankings may facilitate individualized selection of ONB reconstruction techniques in clinical practice.

**Systematic review registration:**

https://www.crd.york.ac.uk/PROSPERO/view/CRD420251143294, identifier CRD420251143294.

## 1 Introduction

Bladder cancer (BC) is among the most common urological malignancies, ranking among the top ten worldwide, with approximately 573,000 new cases and 213,000 deaths annually (1–3). The standard treatment for muscle-invasive bladder cancer (MIBC) and high-risk nonmuscle-invasive bladder cancer (NMIBC) is radical cystectomy (RC), with pelvic lymph node dissection and urinary diversion (4, 5). Among the multiple diversion options, the orthotopic neobladder (ONB) has become a preferred choice, as it utilizes gastrointestinal segments to create a reservoir anastomosed to the urethra, thereby preserving near-physiological voiding function and improving quality of life compared with ileal conduit or cutaneous ureterostomy (6–8).

In recent decades, numerous ONB reconstruction techniques have been developed, such as the Studer pouch, Hautmann pouch, and Mainz pouch (9–11). Although these techniques share the common goal of optimizing ONB function, comparative data on their efficacy remain scarce. Furthermore, conclusions across some studies are inconsistent. Traditional meta-analyses aim to address these limitations, but they remain constrained by their inability to compare multiple interventions simultaneously, as they evaluate only direct comparisons between two techniques and cannot integrate indirect evidence (12). This issue is particularly evident in studies on ONB, where direct head-to-head comparisons between many surgical techniques remain unavailable.

Network meta-analysis (NMA) overcomes these limitations by constructing a network of evidence that includes both direct and indirect comparisons, enabling simultaneous ranking of multiple interventions on the basis of a single outcome (13, 14). To date, no NMA has systematically synthesized data on ONB reconstruction techniques with neobladder functional outcomes as the primary endpoint; even traditional meta-analyses focusing on this topic remain scarce. Therefore, the aim of this study was to fill this gap and provide evidence-based guidance for clinical decision-making in ONB reconstruction.

## 2 Methods

### 2.1 Literature search

This systematic review and NMA were conducted in accordance with the PRISMA extension statement for NMAs to ensure transparent reporting and enable cross-intervention comparisons (15, 16). A comprehensive literature search was conducted in PubMed, EMBASE, and the Cochrane Library from database inception to October 2025. Broadly defined Medical Subject Headings (MeSH) terms were used to identify relevant studies, including “bladder neoplasms”, “urinary diversion”, “orthotopic neobladder”, “cohort studies”, “randomized controlled trials”, and “retrospective studies”. Additional topic-specific free-text terms were incorporated. No restrictions were imposed on the publication date or language. The reference lists of all included studies were manually searched to identify any additional eligible publications. The study protocol was registered in PROSPERO before data extraction.

### 2.2 Inclusion and exclusion criteria

Studies that met the following criteria were included in the NMA:

1. Population: patients diagnosed with BC who underwent RC;
2. Intervention/Control: any type of ONB reconstruction technique**;**
3. Outcomes: at least one reported core functional outcome, including maximum neobladder capacity (MNC), maximal flow rate (MFR), postvoid residual volume (PVR), daytime continence (DC), and nighttime continence (NC) (17).

Two investigators independently screened the studies, with discrepancies resolved through consultation with a third investigator to reach a consensus. For studies with missing or incomplete data, the original author was contacted via email for clarification.

### 2.3 Data extraction and quality evaluation

**Figure 1:**
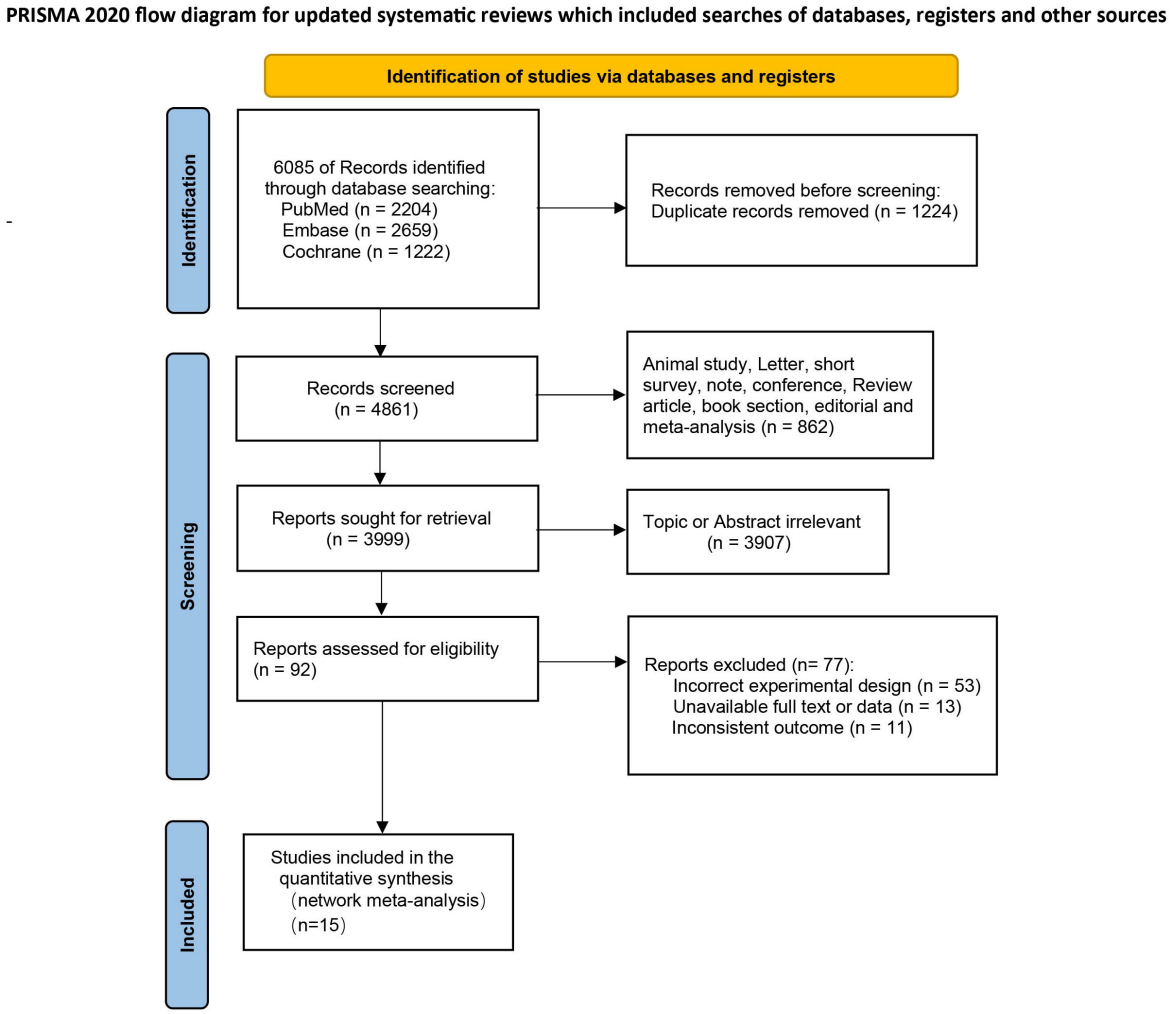
PRISMA flow diagram of the literature search and study selection process.

Two reviewers independently screened 6,085 articles according to the process shown in the PRISMA diagram (**Figure 1**). One reviewer extracted the data, while another verified their accuracy. The extracted information was organized into four domains as follows: study characteristics (author, publication year, and study design), population demographics (sample size, age, and sex distribution), interventions (types of ONB reconstruction techniques), and functional outcomes (MNC, MFR, PVR, DC, and NC) (**Table 1**). For daytime and nighttime continence, most included studies defined satisfactory continence as the use of no more than one pad per day or night. However, postoperative assessment time points and evaluation methods were not fully standardized across studies. The latter included patient self-report, questionnaires, and telephone follow-up.

**Table 1:** Characteristics of studies included in the network meta-analysis.

Study				Population			Outcome				
Author	Year	Study design	Type of neobladder	Sample size, n	Age, mean (±SD)	Sex, (M/F)	MNC (ml), mean (±SD)	MFR (ml/s), mean (±SD)	PVR (ml), mean (±SD)	DC, n (%)	NC, n (%)
Boonchai S. (22)	2023	RCS	Studer-pouch	54	62.5 (±10.7)	54/0	428.9 (±25)	–	50.7 (±18.2)	36 (66.67)	26 (72.22)
			Y-pouch	36	62.7 (±11.9)	28/8	417.2 (±30.6)	–	26.7 (±11.9)	26 (48.15)	20 (55.56)
Lee K.S. (23)	2003	RCS	Studer-pouch	84	–	–	–	–	–	72 (85.71) (n=84)	59 (70.24) (n=84)
			Hautmann-pouch	36	–	–	–	–	–	32(88.89) (n=36)	28 (77.78) (n=36)
Miyake H. (24)	2008	RCS	Studer-pouch	36	–	31/5	–	18.5 (±12.1)	68.7 (±131.3)	–	–
			Hautmann-pouch	9	–	8/1	–	14.5 (±10.8)	48.8 (±68.1)	–	–
			Mainz-pouch	15	–	13/2	–	16.9 (±11.1)	27.3 (±32.3)	–	–
			Goldwasser-pouch	10	–	10/0	–	11.2 (±6.6)	91 (±178)	–	–
			Reddy-pouch	19	–	16/3	–	16.6 (±8.8)	23.7 (±51.9)	–	–
Osman Y. (25)	2004	RCT	Hautmann-pouch	20	–	–	606 (±155)	–	–	–	–
			Kock-pouch	19	–	–	572 (±132)	–	–	–	–
Fakhr I. (26)	2013	RCS	Ileocecal-pouch	61	56.3 (±5.8)	61/0	527.4 (±50.2)	–	–	–	–
			Indiana-pouch	30	54.4 (±4.8)	26/4	481.9 (±73)	–	–	–	–
Zhao Q.X. (27)	2021	RCS	Studer-pouch	23	53.52 (±11.71)	16/7	–	20.1 (±8.5)	–	17 (100) (n=17)	15 (88.24) (n=17)
			Xing-pouch	41	59.2 (±9.18)	36/5	–	20.1 (±3.8)	–	36 (94.74) (n=38)	31 (81.58) (n=38)
Talat Z. (28)	2018	RCS	Studer-pouch	16	58.0 (±4.9)	15/1	–	–	–	14 (87.5)	9 (56.25)
			Anatolian-pouch	36	54.7 (±11.6)	34/2	–	–	–	32 (88.89)	20 (55.56)
Schrier B.P. (29)	2012	RCS	Hautmann-pouch	19	–	–	581.4 (±246)	23.8 (±8.1)	–	–	–
			Reddy-pouch	34	–	–	299.3 (±116.2)	17.2 (±6)	–	–	–
Chen Z.W. (30)	2009	RCT	Studer-pouch	38	65	38/0	385 (±85.4)	11.7 (±6.2)	35.6 (±22.1)	36 (94.74)	32 (84.21)
			Le Bag-pouch	33	61	33/0	405 (±94.6)	13.5 (±5)	32 (±20.5)	31 (93.94)	18 (54.55)
Kulkarni J.N. (31)	2003	RCS	Hautmann-pouch	33	–	33/0	–	–	–	32 (96.97)	29 (87.88)
			Mainz-pouch	35	–	35/0	–	–	–	32 (91.43)	31 (88.57)
			Reddy-pouch	34	–	34/0	–	–	–	27 (79.41)	20 (58.82)
Khafagy M. (32)	2006	RCT	Studer-pouch	29	50	27/2	482 (±221)	–	90.1 (±72.5)	–	–
			Ileocecal-pouch	31	51	28/3	282 (±121)	–	12.2 (±20.3)	–	–
Miyake H. (33)	2012	RCS	Studer-pouch	12	–	0/12	299.7 (±141.5)	19 (±10.9)	62 (±48.9)	–	–
			Reddy-pouch	18	–	0/18	364.2 (±158.7)	21.2 (±13.9)	15.7 (±38.4)	–	–
Paananen I. (34)	2014	PCS	Studer-pouch	30	58 (40-80)	30/0	521.5 (±140.4)	–	–	–	–
			Hautmann-pouch	24	55 (39-71)	24/0	472 (±102.2)	–	–	–	–
			T-pouch	12	63 (53-75)	12/0	626.5 (±114.4)	–	–	–	–
			Goldwasser-pouch	12	62 (52-71)	12/0	579.3 (±111)	–	–	–	–
Hui Z. (35)	2008	RCS	Hautmann-pouch	87	65 (43-74)	–	550 (±122)	18 (±2.6)	82 (±9.4)	87 (100)	79 (90.8)
			Reddy-pouch	65	62 (51-72)	–	420 (±81)	17.5 (±2.2)	79 (±11.5)	61 (93.85)	55 (84.62)
Carini M. (36)	1992	PCS	Studer-pouch	21	54.2 (39-71)	–	311.3 (±39.7)	–	–	21 (100)	12 (57.14)
			Camey I-pouch	17	60.4 (41-74)	–	257.7 (±89.1)	–	–	9 (52.94)	0 (0)

RCS, retrospective cohort study; RCT, randomized controlled trial; PCS, prospective cohort study; M, male; F, female; MNC, maximum neobladder capacity; MFR, maximal flow rate; PVR, postvoid residual volume; DC, daytime continence; NC, nighttime continence; SD, standard deviation.

Continuous variables were expressed as means with standard deviations (SDs) or medians with interquartile ranges (IQRs). When necessary, medians and IQRs were converted to means and SDs using the method described by Hozo et al. (18). Categorical variables were expressed as frequencies and percentages. The methodological quality of each study was independently assessed by two reviewers using design-specific tools. The Cochrane Collaboration’s risk of bias tool was used to evaluate randomized controlled trials (RCTs) in the following six domains: selection bias, performance bias, detection bias, attrition bias, reporting bias, and other types of bias (19, 20). For cohort studies, the Newcastle–Ottawa Scale (NOS), which rates studies on the basis of a star scoring system, was used (21). Any discrepancies were resolved through discussion with a third reviewer.

### 2.4 Statistical analysis

All analyses were conducted within a Bayesian framework using R (Version 4.3.3) and RevMan (Version 5.3). R packages, including gemtc, netmeta, and ggplot2, were used for model fitting, calculation of results, and graph visualization. RevMan was used for risk-of-bias assessment. For continuous variables, comparisons between interventions are presented as mean differences (MDs) and 95% credible intervals (CrIs); for categorical variables, comparisons are reported as risk ratios (RRs) and 95% CrIs. A random-effects model was applied to account for between-study heterogeneity arising from variations in populations, interventions, and follow-up durations. Heterogeneity was quantified using I² (I² > 50% indicated substantial heterogeneity).

To evaluate model consistency, both consistency and inconsistency models were fitted. Under a Bayesian framework, analyses were conducted using the Markov chain Monte Carlo (MCMC) method with three to four chains, 20,000 burn-in iterations and 50,000 iterations. We compared the deviance information criterion (DIC) values between the two models. Because the difference in DIC was less than 5, indicating negligible inconsistency, a consistency model was adopted for subsequent analyses.

Network plots were generated to visualize the connectivity of the comparison network among different ONB reconstruction techniques. Forest plots were produced to display pairwise comparisons relative to a specified reference technique. Model convergence was assessed using trace and density plots. League tables were used to summarize the results of all pairwise comparisons, while cumulative ranking and ranking probability plots were used to evaluate the relative efficacy of each technique. Local inconsistency tests were performed using the node-splitting method, and global heterogeneity was assessed with corresponding forest plots. For all analyses, P < 0.05 was considered to indicate statistical significance.

## 3 Results

### 3.1 Search results

In accordance with the predefined search strategy, we initially identified 6,085 records from three electronic databases. After removing 1,224 duplicates, we further excluded 4,846 ineligible studies on the basis of the established criteria. Ultimately, 15 studies that met all the inclusion criteria were included in the NMA (22–36). The detailed screening process is presented in **Figure 1**.

### 3.2 Characteristics of the included studies

The baseline characteristics are summarized in **Table 1**. The included studies, published between 1992 and 2023, comprised 3 RCTs and 12 cohort studies. The analysis included 1,129 participants and directly compared 14 distinct ONB reconstruction techniques—the Studer pouch, Hautmann pouch, Y pouch, T pouch, Xing pouch, Kock pouch, Camey I pouch, Mainz pouch, Le Bag pouch, Goldwasser pouch, Reddy pouch, Ileocecal pouch, Indiana pouch, and Anatolian pouch.

**Figure 2:**
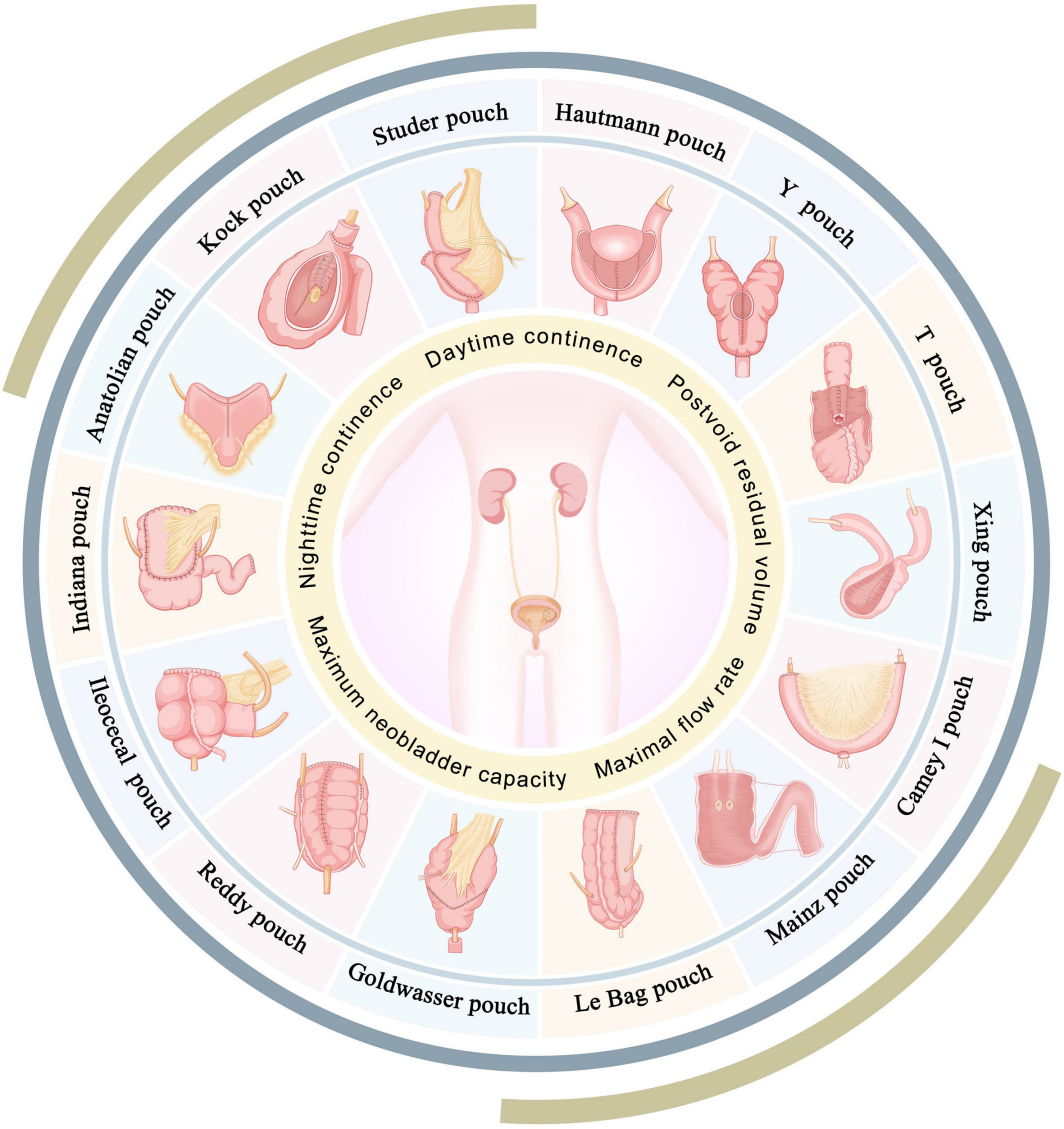
Schematic overview of 14 orthotopic neobladder (ONB) reconstruction techniques and five core functional outcomes. This image illustrates the anatomical configurations of the 14 ONB reconstruction techniques included in the network meta-analysis, arranged circumferentially. The inner ring summarizes the five predefined core functional outcomes evaluated across techniques.

As illustrated in **Figure 2**, the included ONB techniques exhibit substantial heterogeneity in anatomical configuration, which may reflect diverse surgical strategies tailored to optimize different aspects of postoperative function. This figure provides a structural framework for interpreting the subsequent comparative evaluation of core functional outcomes across these ONB techniques. The predefined core functional outcomes included MNC, MFR, PVR, DC, and NC.

### 3.3 Quality assessment

The risk of bias assessment results for the 3 RCTs are presented in **Supplementary Figures 1A, B**. All trials used clear randomization methods for participant allocation. Allocation concealment was reported in 2 trials. No trials reported blinding measures for participants, researchers, or outcome assessors. Two trials had no or adequately managed attrition. One trial had a high dropout rate (>20%) without adequate explanation; thus, it was rated “high risk”. All 3 RCTs showed consistency between prespecified and reported outcomes. No additional biases were identified.

The 12 cohort studies were assessed using the NOS, with all achieving scores of 6–8, indicating overall high methodological quality (**Supplementary Figure 1C**).

### 3.4 Astringency and inconsistency evaluation

Relevant Gelman–Rubin diagnostic plots, trace plots, and density plots are provided in the **Supplementary Material**. Gelman–Rubin diagnostic plots confirmed the convergence of the MCMC methods. For nearly all pairwise comparisons, the median and 97.5% quantile values of the potential scale reduction factor (PSRF) rapidly decreased to approximately 1 within the first 20,000 iterations and remained stable thereafter (total iterations set at 50,000), with minimal within-chain variation. Trace plots showed stable oscillations after the burn-in period, and density plots demonstrated largely symmetric, unimodal posterior distributions. Collectively, these results validated the robustness of the parameter estimates.

The only exception was the comparison between the Studer pouch and Camey I pouch for NC. For this comparison, the 97.5% quantile of the PSRF fluctuated, and the trace plot displayed broader variation. Moreover, the density plot revealed a skewed distribution. Heterogeneity analyses can be found in **Supplementary Figures 5**, **10**, **15**, **19**, and **24**.

### 3.5 Network meta-analysis

#### 3.5.1 Comparison of maximum neobladder capacities

**Figure 3:**
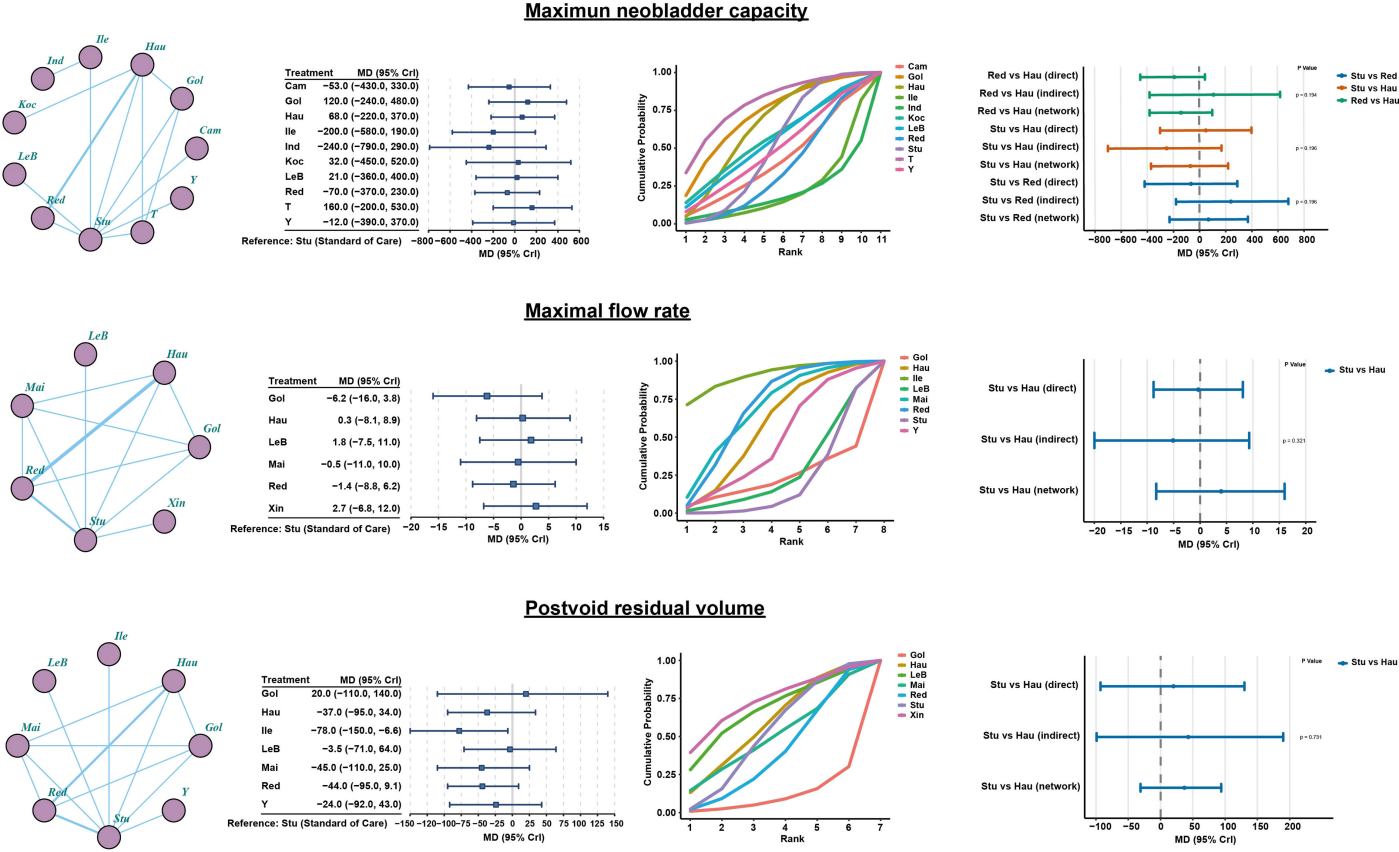
Primary continuous outcomes of the network meta-analysis. This image summarizes the comparative performance of the included orthotopic neobladder (ONB) techniques across three continuous functional outcomes: maximum neobladder capacity (MNC), maximal flow rate (MFR), and postvoid residual volume (PVR). For each outcome, the first panel depicts the network plot, with nodes representing ONB techniques and edges indicating available direct comparisons. The second panel presents the forest plot of pairwise comparisons derived from a random-effects Bayesian model. The third panel displays the cumulative ranking curves of each technique. The fourth panel summarizes the node-splitting analysis used to assess local inconsistency. MNC, maximum neobladder capacity; MFR, maximal flow rate; PVR, postvoid residual volume; Ile, Ileocecal pouch; Hau, Hautmann pouch; Gol, Goldwasser pouch; Cam, Camey I pouch; Y, Y pouch; T, T pouch; Stu, Studer pouch; Red, Reddy pouch; LeB, Le Bag pouch; Koc, Kock pouch; Ind, Indiana pouch; Xin, Xing pouch; Mai, Mainz pouch; MD, mean difference; CrI, credible interval.

Ten studies reported the MNC for 11 ONB reconstruction techniques. The corresponding evidence network is illustrated in **Figure 3**. In this analysis, a greater MNC (within physiological limits) was considered favorable. No statistically significant differences were found between the Studer pouch and any of the other 10 techniques (P > 0.05; **Figure 3**). However, the cumulative ranking and ranking probability plots suggested that among the 11 included techniques, the T pouch exhibited the best overall efficacy in improving MNC, whereas the Indiana pouch exhibited the worst performance (**Figure 3**). The 11 ONB reconstruction techniques were ranked based on MNC as follows (from best to worst efficacy): T pouch, Goldwasser pouch, Hautmann pouch, Kock pouch, Le Bag pouch, Studer pouch, Y pouch, Camey I pouch, Reddy pouch, Ileocecal pouch, and Indiana pouch (**Figure 3**; **Supplementary Figure 4**). Local inconsistency was assessed using the node-splitting method, which revealed no statistically significant differences between direct and indirect comparisons (P > 0.05; **Figure 3**). The league table is provided in **Supplementary Figure 6**. Owing to limitations in sample size and data availability, local inconsistency tests were feasible only for partial pairwise comparisons.

#### 3.5.2 Comparison of maximal flow rates

Six studies reported MFR data for 7 ONB reconstruction techniques; the corresponding evidence network is illustrated in **Figure 3**. Consistent with the criterion applied for MNC, a greater MFR was considered favorable following ONB reconstruction. No statistically significant differences were detected between the Studer pouch and any of the other 6 techniques (P > 0.05; **Figure 3**). Cumulative ranking and ranking probability plots indicated that the Xing pouch demonstrated the best comprehensive efficacy in improving MFR, whereas the Goldwasser pouch had the lowest efficacy (**Figure 3**). The overall ranking of the 7 ONB reconstruction techniques was illustrated as follows (with highest to lowest efficacy): Xing pouch, Le Bag pouch, Hautmann pouch, Studer pouch, Mainz pouch, Reddy pouch, and Goldwasser pouch (**Figure 3**; **Supplementary Figure 9**). No significant local inconsistencies were detected (P > 0.05; **Figure 3**). The details of the league table are presented in **Supplementary Figure 11**. Owing to the limited data, inconsistency assessments could be conducted for only a subset of comparisons.

#### 3.5.3 Comparison of postvoid residual volumes

For PVR, data from 6 studies were synthesized, and 8 ONB reconstruction techniques were compared. The corresponding evidence network is shown in **Figure 3**. In contrast to the evaluation criteria used for MNC and MFR, a lower PVR was considered favorable. Pairwise comparisons between the Studer pouch and other techniques revealed no statistically significant difference (P > 0.05), except for the Ileocecal pouch, which had a significantly lower PVR than the Studer pouch (P < 0.05; **Figure 3**). Cumulative ranking and ranking probability plots further supported these findings, indicating that the Ileocecal pouch had the highest probability of achieving the most favorable PVR, whereas the Studer pouch had the lowest probability (**Figure 3**). The overall ranking of the 8 ONB reconstruction techniques was presented as follows (from most to least favorable): Ileocecal pouch, Reddy pouch, Mainz pouch, Hautmann pouch, Y pouch, Le Bag pouch, Goldwasser pouch, and Studer pouch (**Figure 3**; **Supplementary Figure 14**). The results of local inconsistency tests revealed no statistically significant differences (P > 0.05; **Figure 3**). The relevant league table is provided in **Supplementary Figure 16**. Owing to limited sample sizes and data availability, inconsistency tests could not be applied to all pairwise comparisons.

#### 3.5.4 Comparison of daytime continence

**Figure 4:**
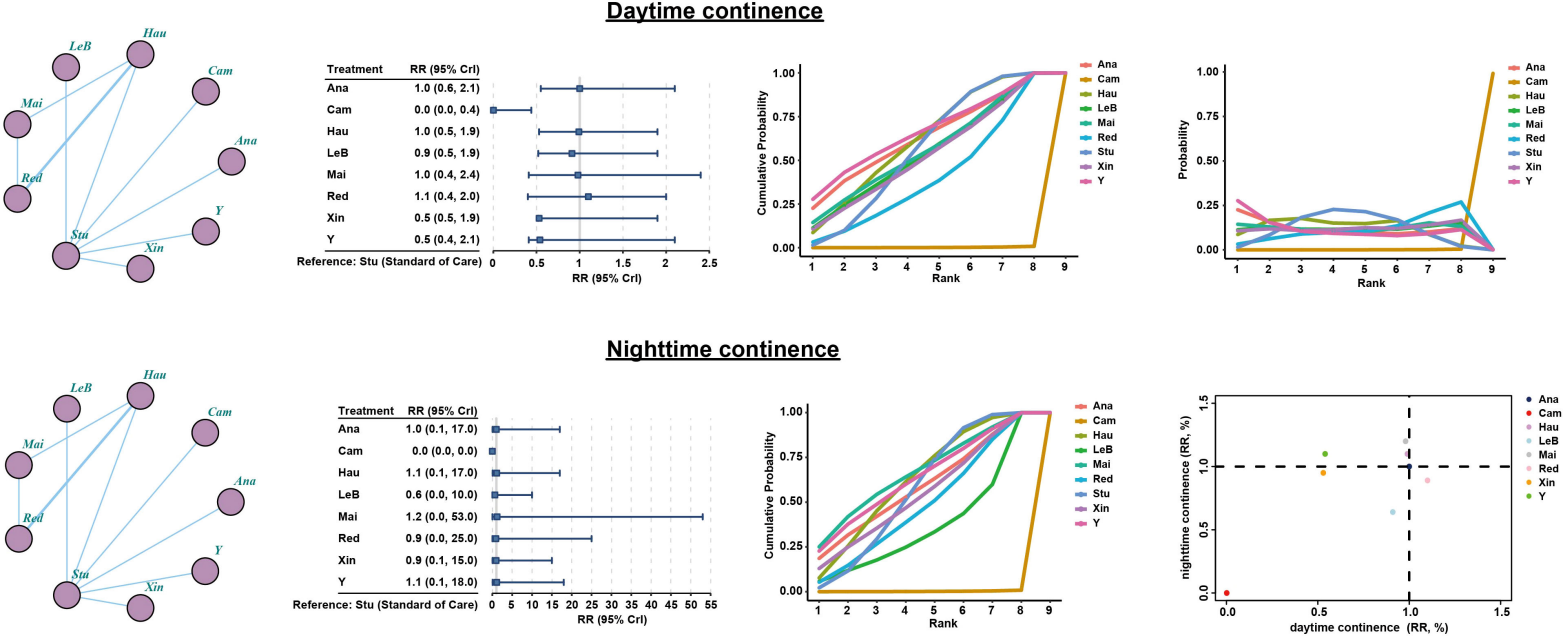
Primary categorical outcomes of the network meta-analysis. Each row represents one of the two categorical functional outcomes: daytime continence (DC), and nighttime continence (NC). For each outcome, the first panel depicts the network plot, with nodes representing orthotopic neobladder (ONB) techniques and edges indicating available direct comparisons. The second panel provides the forest plot of pairwise comparisons obtained from a random-effects Bayesian model. The third panel displays the cumulative ranking curves of each technique. For DC, the fourth panel summarizes the corresponding ranking probability curves. For NC, the fourth panel presents a two-dimensional scatter plot illustrating the distribution of DC and NC across different ONB, using the Studer pouch as the reference, to further explore the relationship between these two outcomes. DC, daytime continence; NC, nighttime continence; Hau, Hautmann pouch; Cam, Camey I pouch; Ana, Anatolian pouch; Y, Y pouch; Xin, Xing pouch; Stu, Studer pouch; Red, Reddy pouch; Mai, Mainz pouch; LeB, Le Bag pouch; RR, risk ratio; Crl, credible interval.

For DC, data from 8 studies were collected to evaluate the 9 ONB reconstruction techniques; the network plot is presented in **Figure 4**. In this analysis, a higher DC rate was considered to indicate a superior functional outcome. As illustrated in the forest plot, the performance of the Studer pouch did not differ significantly from that of most techniques (P > 0.05), but it performed significantly better than the Camey I pouch (P < 0.05; **Figure 4**). Cumulative ranking and ranking probability plots further revealed that the Y pouch was most likely to achieve optimal DC, whereas the Camey I pouch was the least effective (**Figure 4**). The specific rankings were as follows (from highest to lowest probability of achieving optimal DC): Y pouch, Anatolian pouch, Hautmann pouch, Studer pouch, Mainz pouch, Le Bag pouch, Xing pouch, Reddy pouch, and Camey I pouch (**Figure 4**). The league table is displayed in **Supplementary Figure 20**. Owing to the limited sample sizes and data availability, the node-splitting method could not be applied to conduct local inconsistency tests.

#### 3.5.5 Comparison of nighttime continence

The last functional outcome was NC. Consistent with the analytical framework used for DC, data from 8 eligible studies were synthesized to compare the effects of 9 ONB reconstruction techniques. The corresponding evidence network is presented in **Figure 4**. Interpretation of the forest plot indicated that, except for the Camey I pouch, no statistically significant differences were observed between the Studer pouch and any of the other 7 reconstruction techniques (P > 0.05; **Figure 4**). For the comparison between the Studer pouch and Camey I pouch, the RR was close to 0. Cumulative ranking and ranking probability plots revealed that the Mainz pouch had the highest likelihood of achieving optimal NC, whereas the Camey I pouch demonstrated the lowest likelihood, showing significantly inferior performance compared with the other 8 techniques (**Figure 4**). The overall rankings were as follows: Mainz pouch, Y pouch, Hautmann pouch, Anatolian pouch, Studer pouch, Xing pouch, Reddy pouch, Le Bag pouch, and Camey I pouch (**Figure 3**; **Supplementary Figure 23**). The corresponding league table is provided in **Supplementary Figure 25**. Additionally, local inconsistency tests were not feasible. To further explore the relationship between DC and NC, a two-dimensional scatter plot of effect size distributions was constructed using the Studer pouch as the reference. As shown in **Figure 4**, the Y pouch had RRs greater than 1 for both outcomes, indicating potential advantages in maintaining 24-hour continence compared with the Studer pouch. In contrast, the RRs of the Xing and Hautmann pouches were less than 1 for both outcomes, indicating relatively poor continence performance. Other techniques were clustered near the reference lines, implying minimal differences from the Studer pouch. Notably, compared with the Studer pouch, the Camey I pouch displayed an extremely low RR (RR = 0.00099), with an unusually wide CI, warranting cautious interpretation of these results.

## 4 Discussion

Unlike traditional meta-analyses, which are limited to pairwise direct comparisons, NMA enables simultaneous evaluation of multiple interventions by integrating both direct and indirect evidence, thereby constructing a holistic evidence network and allowing systematic comparative ranking of interventions (13, 14). The present study is the first to compare core functional outcomes across various ONB reconstruction techniques, and it strictly adhered to standardized methodological procedures for NMAs (16).

Overall, statistically significant differences were rarely observed among the ONB techniques evaluated in this NMA across the 5 core functional outcomes, particularly when the classic Studer pouch was compared with other techniques (37, 38). These findings are consistent with those of previous studies, indicating that most contemporary reconstruction techniques achieve comparable functional outcomes (23, 39). Despite technical variations or modifications, all ONB techniques are based on the following common reconstructive rationale: detubularizing a segment of the ileum, caecum, or colon; reshaping the segment into a reservoir; and performing ureteral and urethral anastomoses. Moreover, ONB techniques follow identical surgical principles—minimizing anastomotic tension, maintaining a near-spherical reservoir configuration, and ensuring adequate capacity and compliance. Such a shared design and biomechanics likely explain the limited functional differences between the Studer pouch and other ONB techniques.

Interestingly, three key findings emerged from the present analysis and may offer useful guidance for clinical decision-making. First, compared with the Camey I pouch, the Studer pouch demonstrated superior NC (P<0.05); however, the RR was close to 0, with an extremely wide CI. This was attributed to the fact that only one study comparing NC, in which no patients in the Camey I group achieved NC. Second, compared with the Studer pouch, the Ileocecal pouch was significantly superior in terms of PVR. PVR serves as a key indicator of neobladder emptying efficiency and is closely associated with the risk of postoperative complications following ONB reconstruction (40). Among the 8 evaluated techniques, only the Ileocecal pouch demonstrated a significantly lower PVR than the Studer pouch, and a cumulative ranking plot further confirmed the superior efficacy of the Ileocecal pouch. This advantage may stem from the unique anatomical and functional features of the Ileocecal segment: the Ileocecal valve provides an inherent antireflux mechanism, whereas the thicker smooth muscle layer of the caecum facilitates stronger contractility and more complete emptying, thereby reducing PVR (41). Given the greater complexity of the surgical procedure (which requires strict preservation of ileocecal valve integrity), surgeons need substantial experience in intestinal anastomosis (26).

Third, as illustrated by a two-dimensional scatter plot, when DC and NC were jointly evaluated, the Y pouch demonstrated the most favorable performance among the assessed ONB techniques (**Figure 4**). This finding may be explained by the branched configuration of the Y pouch, which reduces tension at the urethral anastomosis, thereby minimizing traction or compression on the urethral sphincter and lowering the incidence of postoperative urinary incontinence. Concurrently, the anastomotic procedure between the ureter and Y pouch is simple and does not require an additional antireflux mechanism, potentially reducing the risk of anastomotic strictures and obviating the need for postoperative vesical fistulization. Together, these advantages may contribute to improved voiding function and continence outcomes (22).

Furthermore, to comprehensively delineate the differences among ONB techniques, we constructed cumulative ranking and ranking probability plots using SUCRA-derived rankings. These visualizations transform complex statistical outputs into clinically interpretable hierarchies, thereby providing intuitive and practical evidence to guide individualized ONB selection. The SUCRA-based rankings for each core functional outcome are summarized in the results.

Local inconsistency assessments and heterogeneity analyses are crucial for NMAs. Accordingly, both were performed in the present study. The results of the node-splitting analysis supported the assumption of local consistency, indicating that direct and indirect comparisons yielded consistent results in specific pairwise comparisons. Owing to the limited number of included studies, inconsistency testing for DC and NC was not feasible. Heterogeneity was quantified using the I² statistic, calculated only for comparisons including at least two studies (42). The heterogeneity analyses indicated substantial variation among some direct comparisons, probably due to small sample sizes, whereas heterogeneity could not be assessed for comparisons based on a single study. Results are presented in **Supplementary Figures 5**, **10**, **15**, **19**, and **24**.

It should be noted that postoperative functional outcomes following RC may depend not only on the choice of neobladder configuration, but also on surgical technique. Previous studies have demonstrated that the combination of the integrated pelvic fascial structure-sparing (IPFSS) technique and intracorporeal ONB reconstruction may facilitate earlier recovery of continence and sexual function in male patients after surgery (43). In pediatric practice, RC combined with detaenial sigmoid neobladder reconstruction has been associated with favorable continence and voiding outcomes while maintaining oncologic control, which has been attributed to standardized stepwise surgical techniques and individualized anatomical adaptation (44). In addition, modified neobladder techniques tailored to female pelvic anatomy have been shown to reduce the risk of postoperative chronic urinary retention in women (45). Collectively, these findings suggest that future studies should also investigate the impact of surgical technical factors on patient outcomes, thereby promoting more precise and individualized approaches to ONB reconstruction.

Although this NMA provides a comprehensive comparison of multiple ONB reconstruction techniques, several limitations should be acknowledged. First, the overall study design was imbalanced, with cohort studies accounting for 80% of the included studies and only three being RCTs. Second, certain ONB techniques were supported by only a single small-sample study, resulting in an unevenly connected evidence network. Additionally, certain ONB techniques, such as the Padovana pouch and Florence pouch, could not be included because the available evidence constituted disconnected networks without shared comparators with the main evidence network (46, 47). Third, although the definition of continence was generally consistent across studies, postoperative assessment time points and evaluation methods varied, which may have introduced measurement heterogeneity into the pooled analyses. Fourth, patients undergoing different ONB techniques may represent highly selected populations with varying surgical indications and baseline characteristics, potentially introducing selection bias and affecting the transitivity assumption underlying NMA. Finally, postoperative ONB function may be influenced by multiple patient- and surgery-related factors beyond neobladder configuration itself, including age, sex, nerve or urethral preservation, surgical approach, and surgeon experience. Although subgroup analyses based on these variables would have been clinically meaningful, the limited number of eligible studies and sparse outcome data may have resulted in substantial heterogeneity and unstable network estimates. Therefore, these factors were not analyzed as subgroup variables, and their potential confounding effects cannot be completely excluded.

## 5 Conclusion

The present NMA included 14 ONB reconstruction techniques and compared their efficiency across 5 core functional outcomes. Overall, most techniques demonstrated comparable performance; however, the Studer pouch showed significantly better NC than the Camey I pouch (P<0.05), while the Ileocecal pouch achieved superior PVR compared with the classic Studer pouch. When DC and NC were jointly assessed, the Y pouch may offer functional advantages among the various ONB techniques. Moreover, SUCRA-based ranking provided clinically meaningful evidence to support the individualized selection of ONB reconstruction techniques. Given the limitations of this study, further large-scale, high-quality clinical studies are warranted to validate these findings. Additionally, the momentum of scientific exploration must be sustained to provide more critical, evidence-based support for optimizing ONB technique selection in clinical practice.

## Data Availability

The original contributions presented in the study are included in the article/**Supplementary Material**. Further inquiries can be directed to the corresponding author.
