# Supplementary material for: Comparison of the efficacy of different orthotopic neobladder reconstruction techniques following radical cystectomy for bladder cancer: a network meta-analysis
Source: Front Oncol. 2026 Jun 10;16:1857787. doi: 10.3389/fonc.2026.1857787 (PMC13290509; doi:10.3389/fonc.2026.1857787)
Supplement: Supplementary file 1 [file DataSheet1.docx]

**Supplementary Figures**

**Supplementary Figure S1
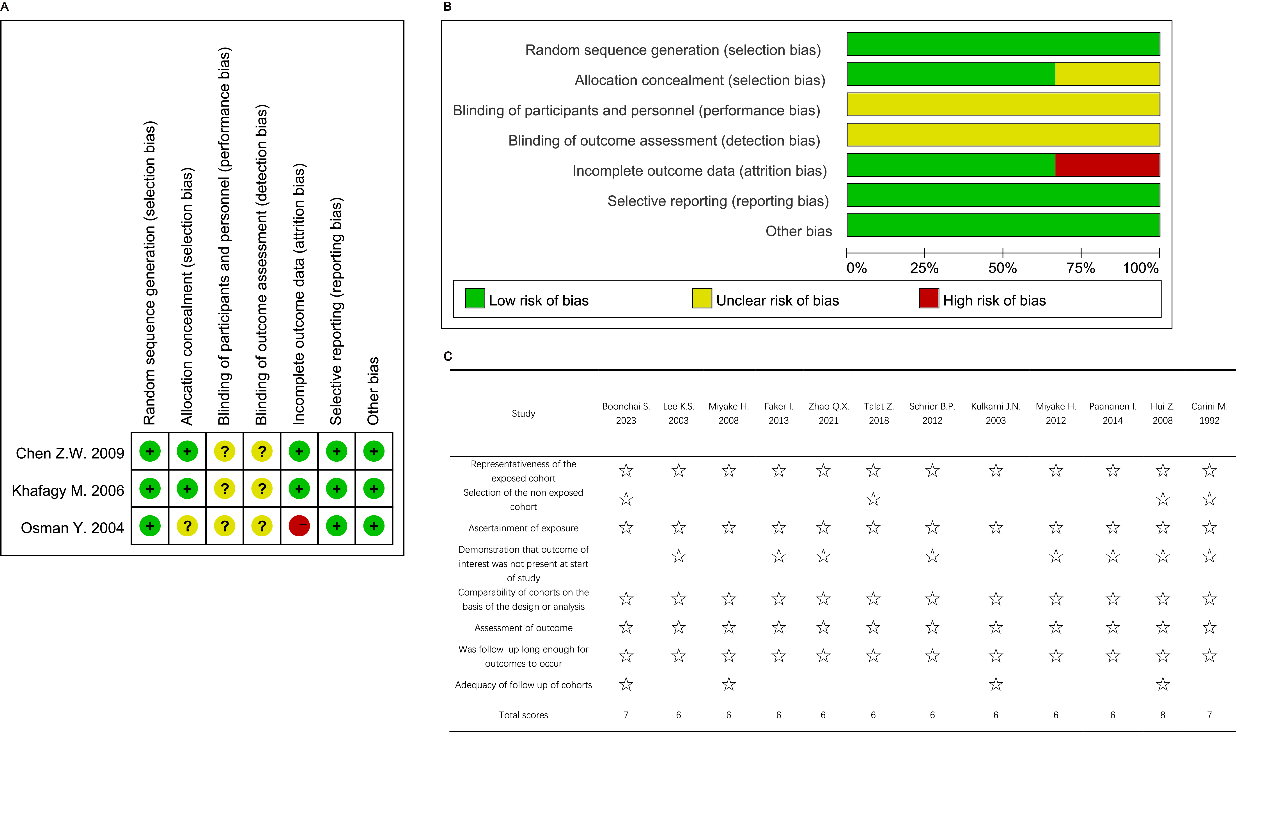
**

**Supplementary Figure S2**

**
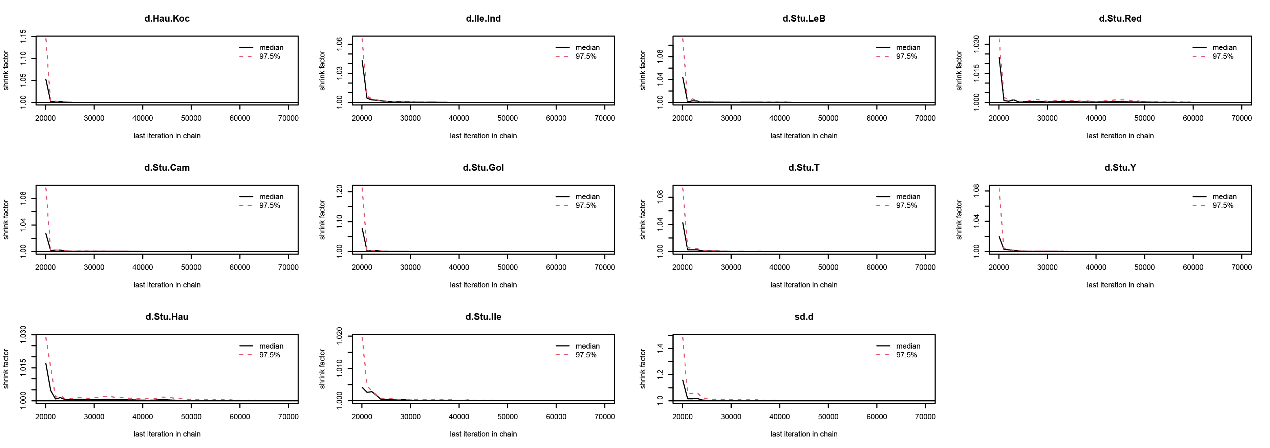
**

**Supplementary Figure S3**

**
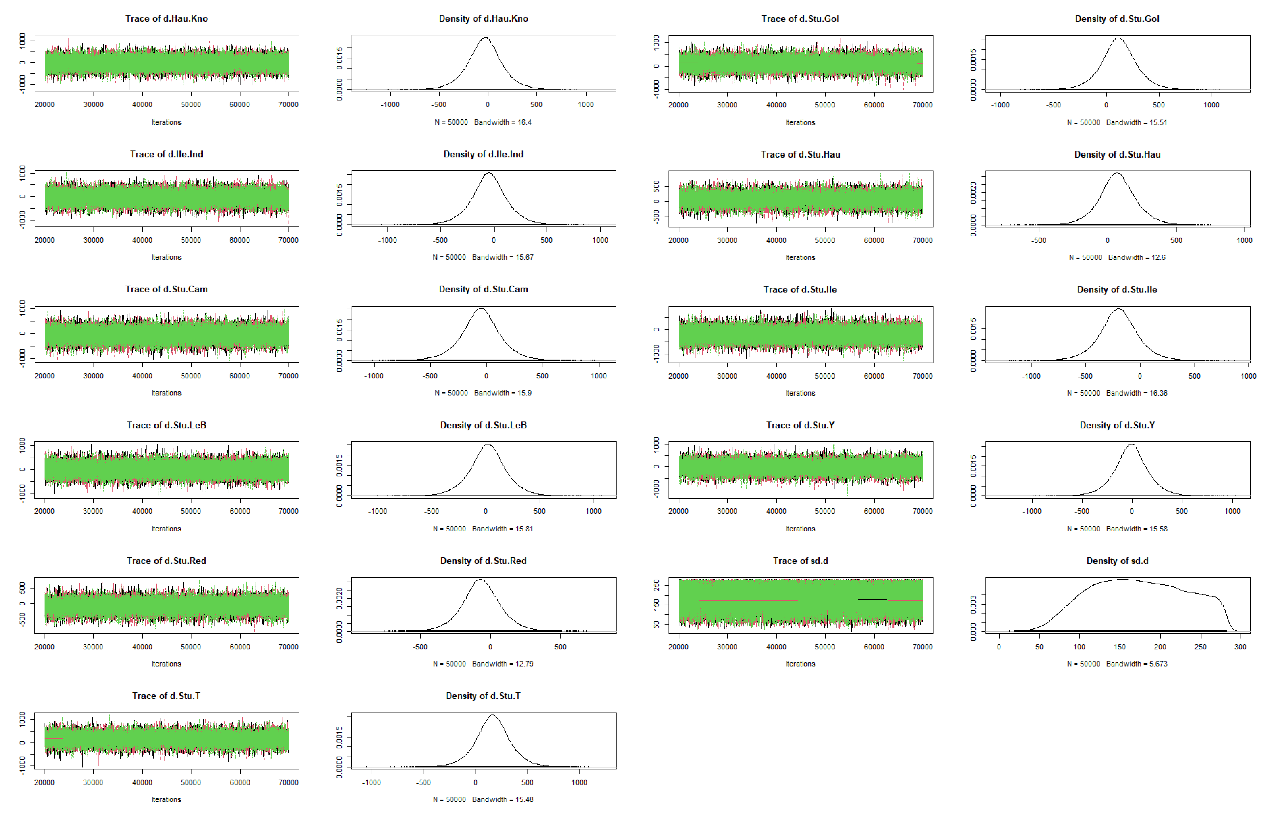
**

**Supplementary Figure S4**

**
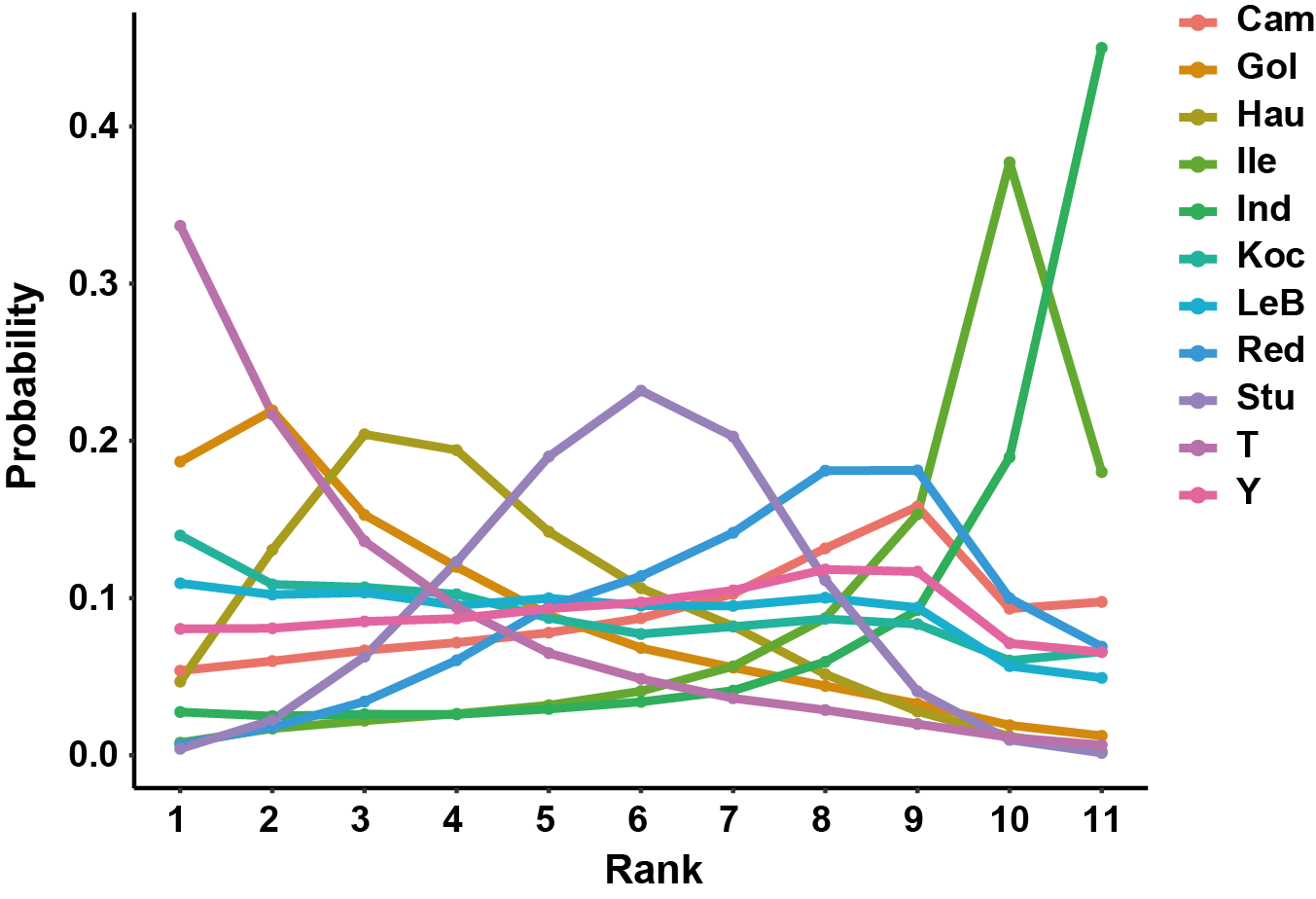
**

**Supplementary Figure S5**
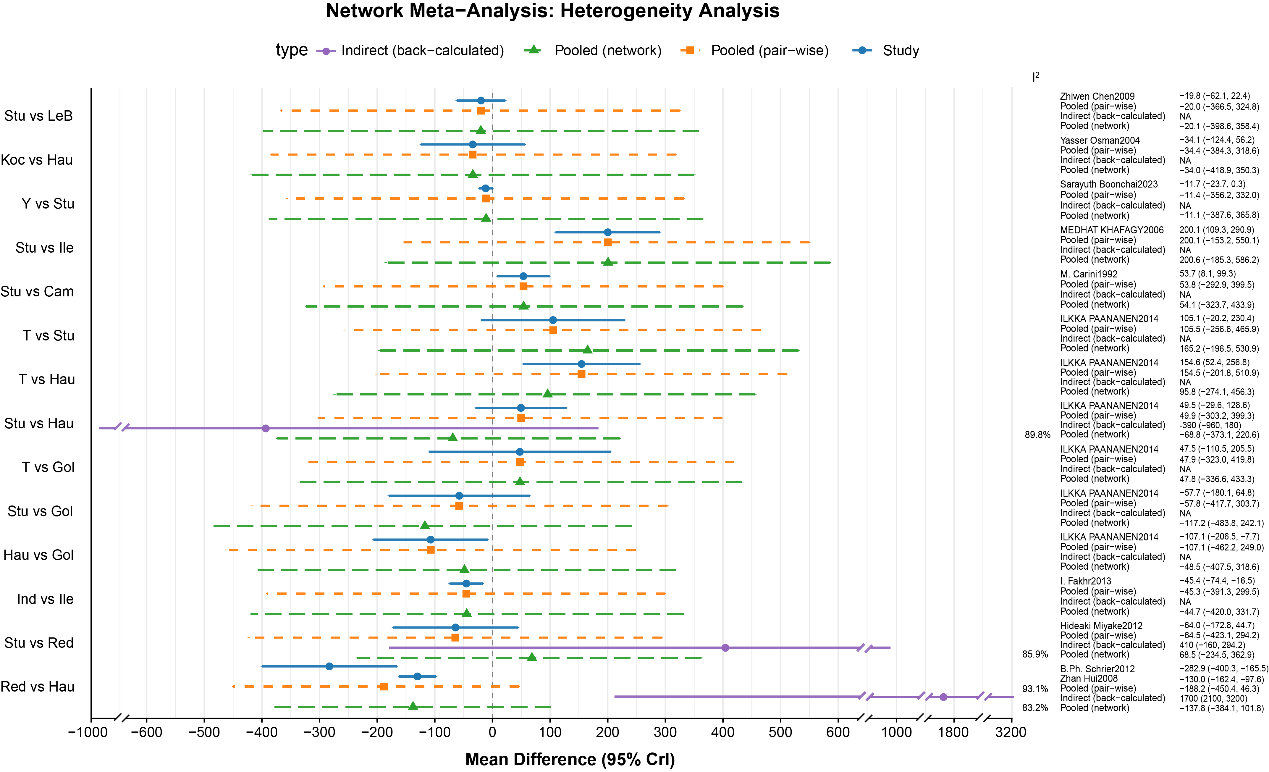


**Supplementary Figure S6**


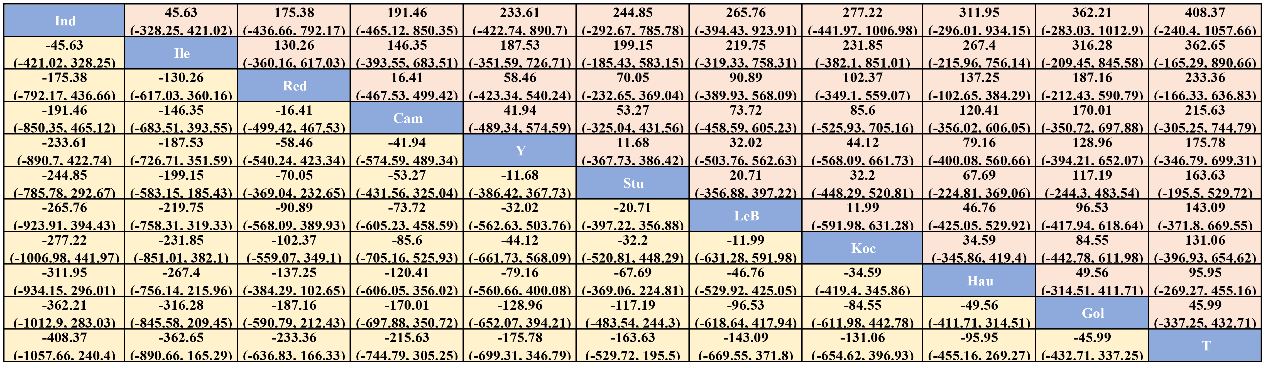


**Supplementary Figure S7**
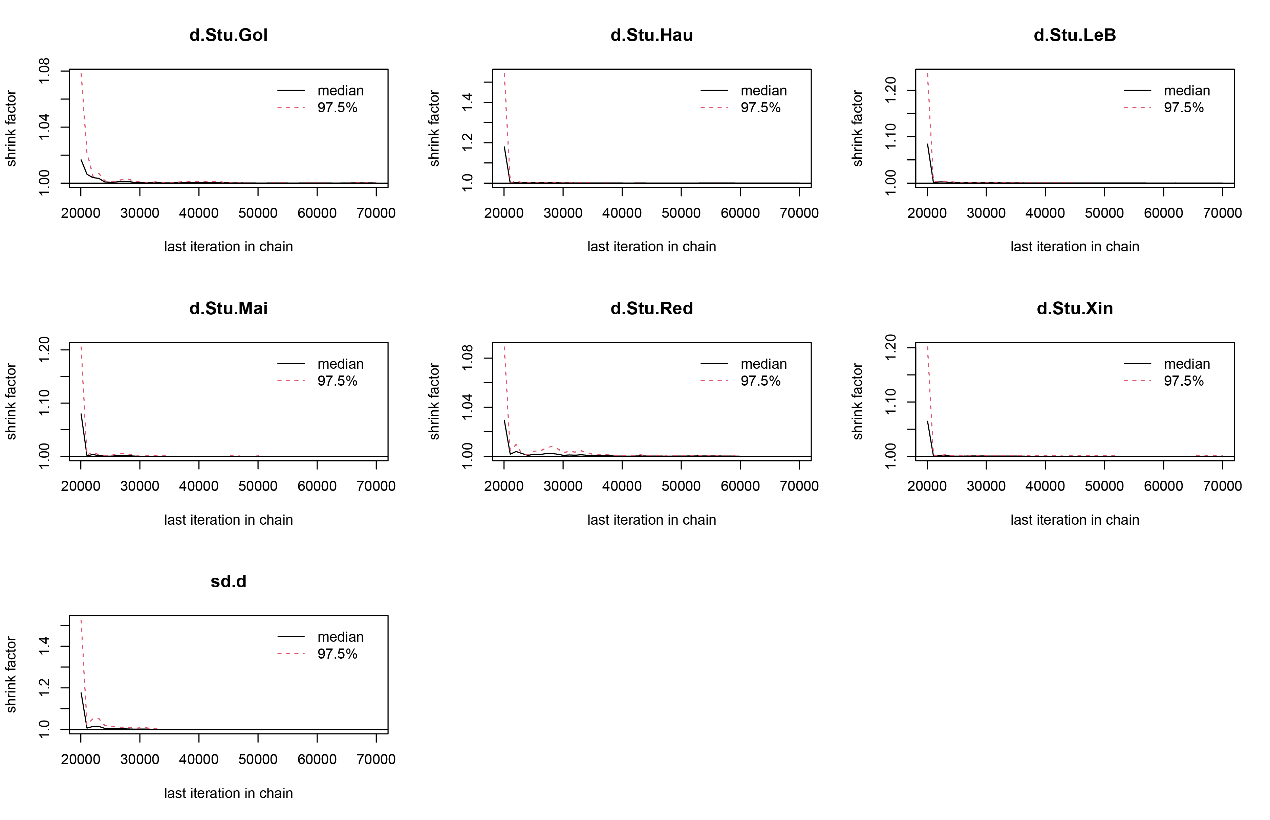


**Supplementary Figure S8**
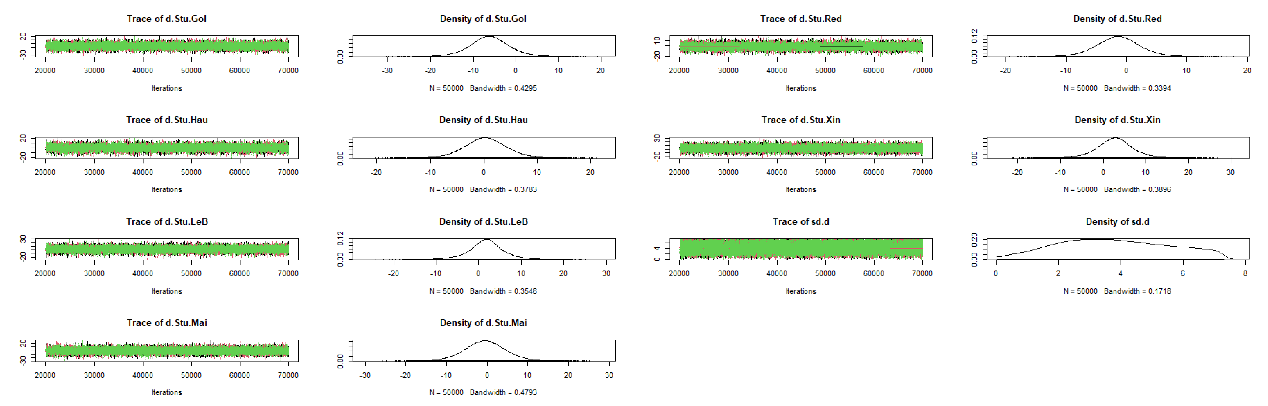


**Supplementary Figure S9**
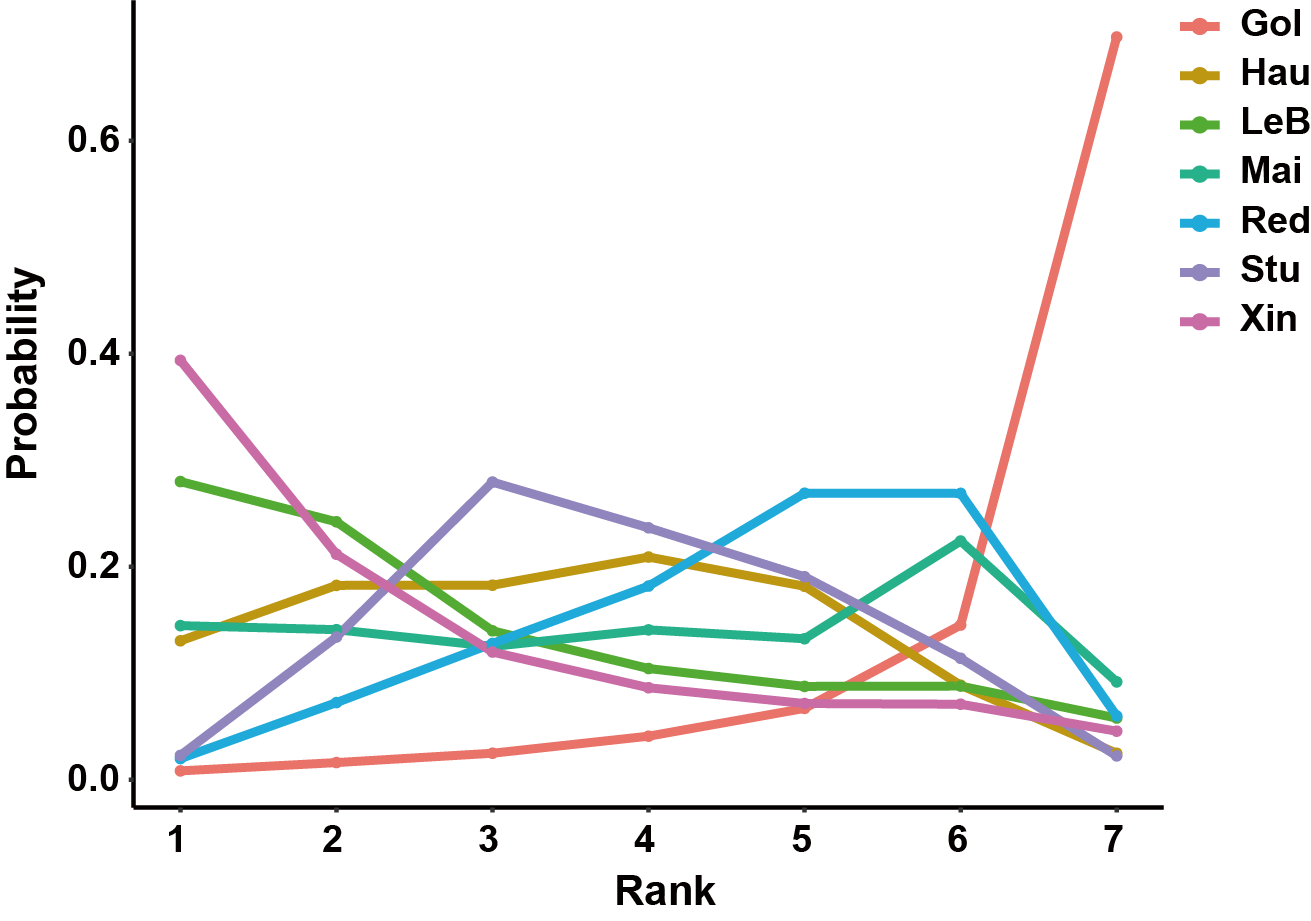


**Supplementary Figure S10**
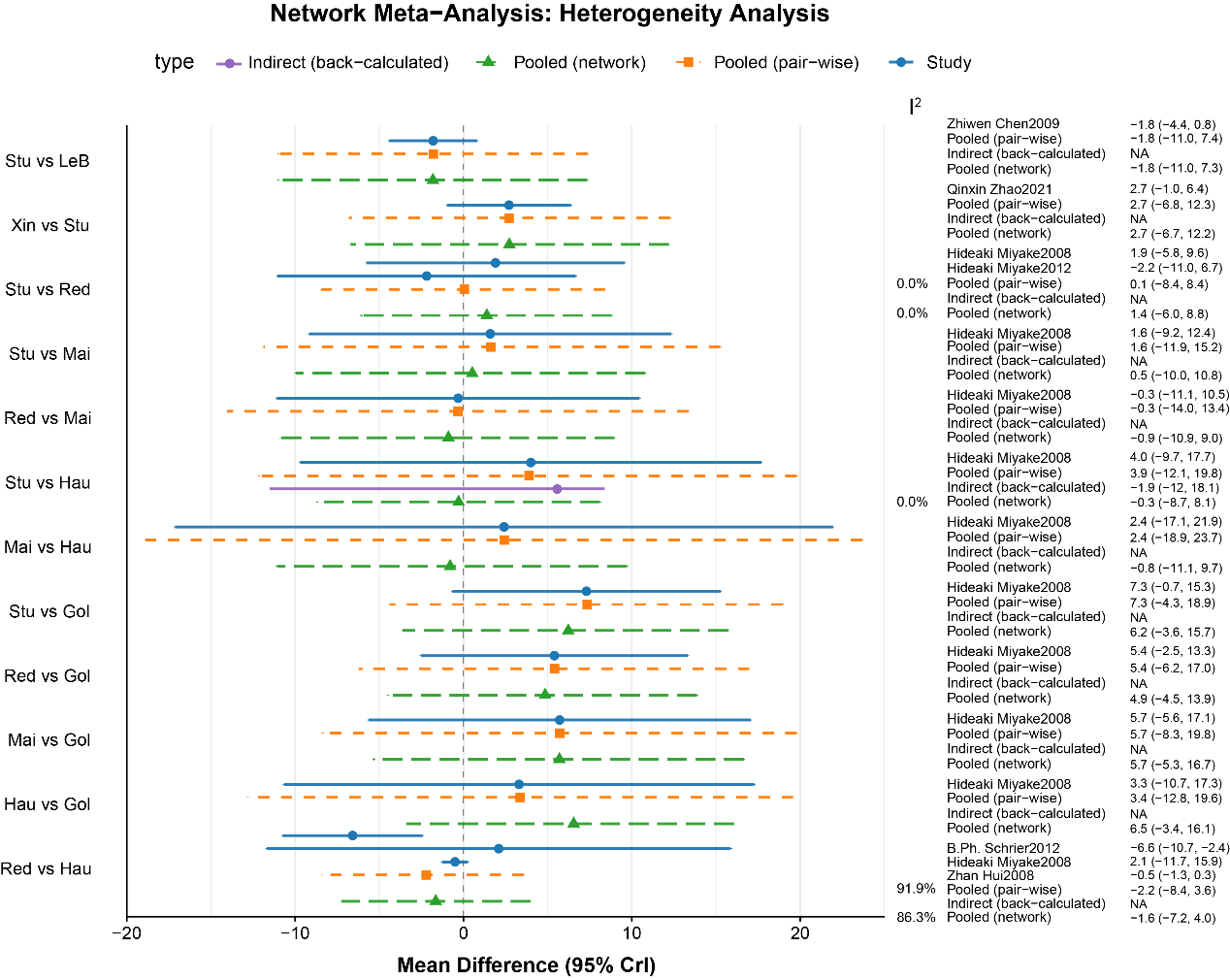


**Supplementary Figure S11**
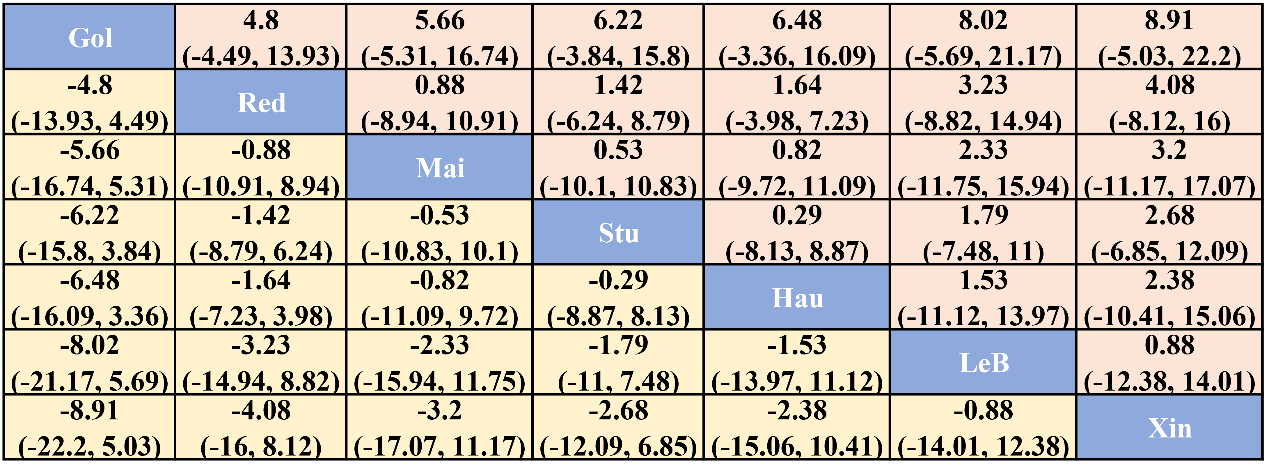


**Supplementary Figure S12**
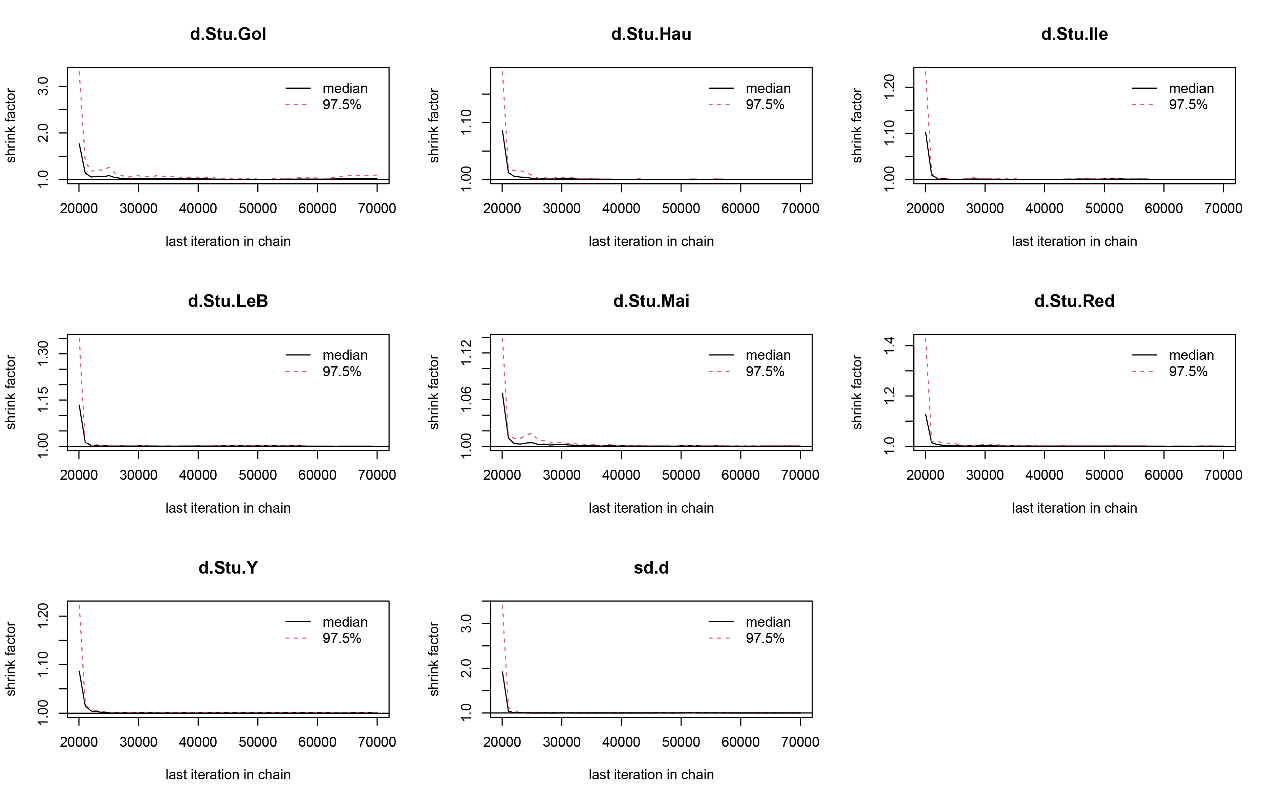


**Supplementary Figure S13**
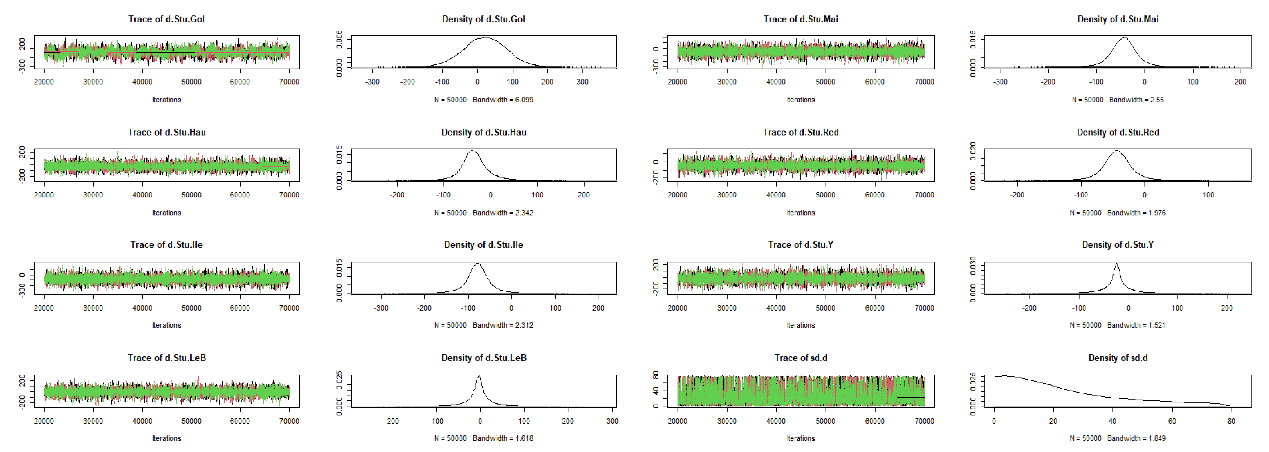


**Supplementary Figure S14**


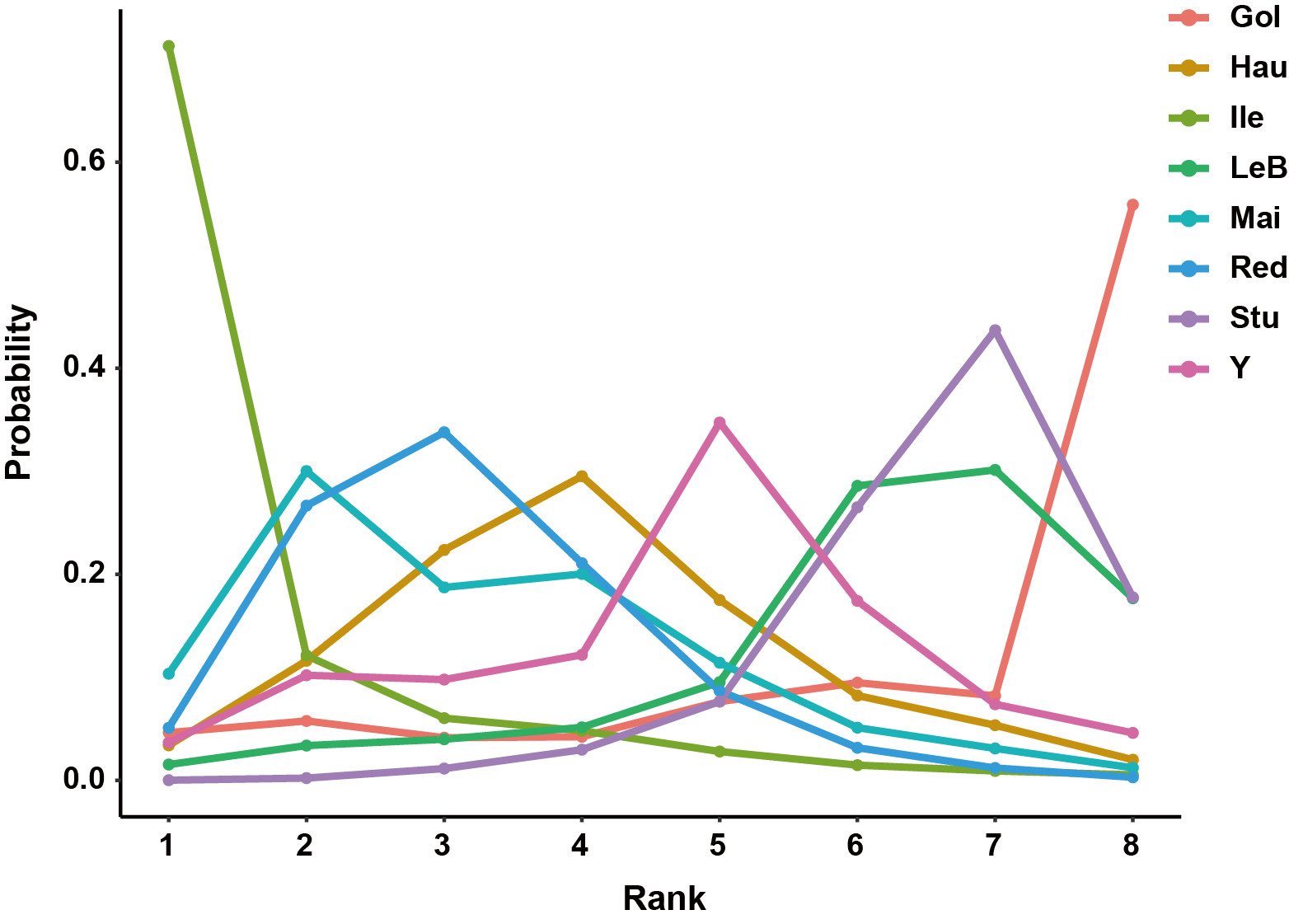


**Supplementary Figure S15**
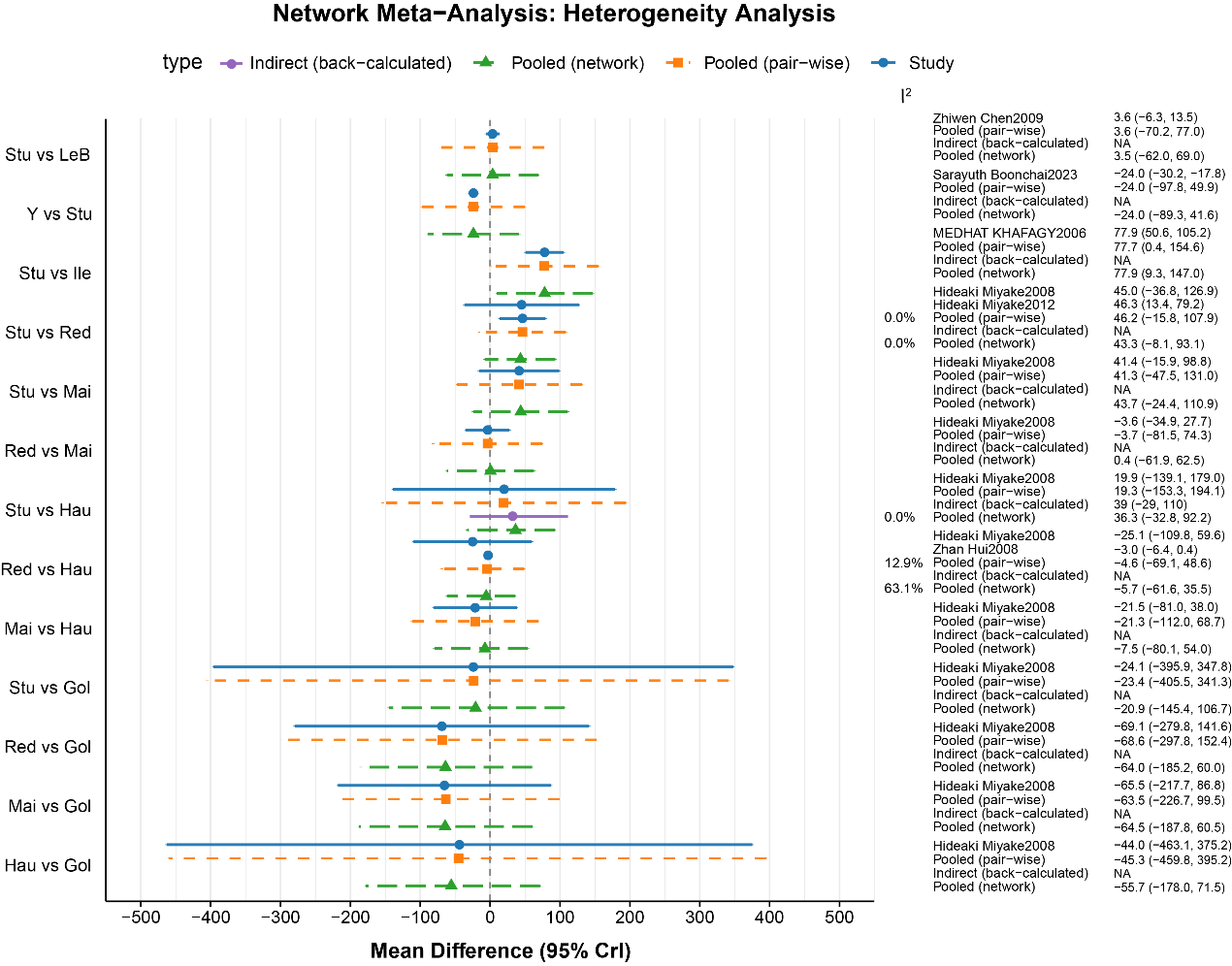


**Supplementary Figure S16**
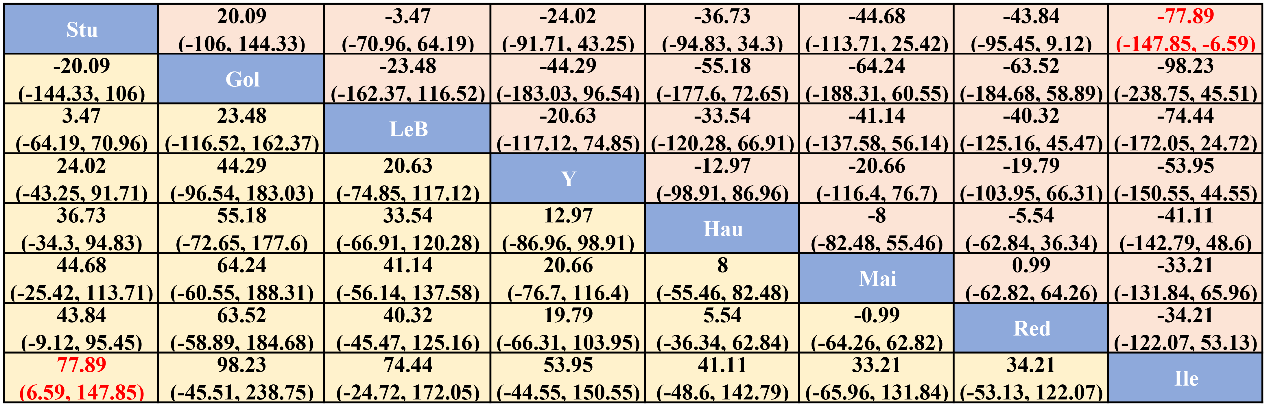


**Supplementary Figure S17**
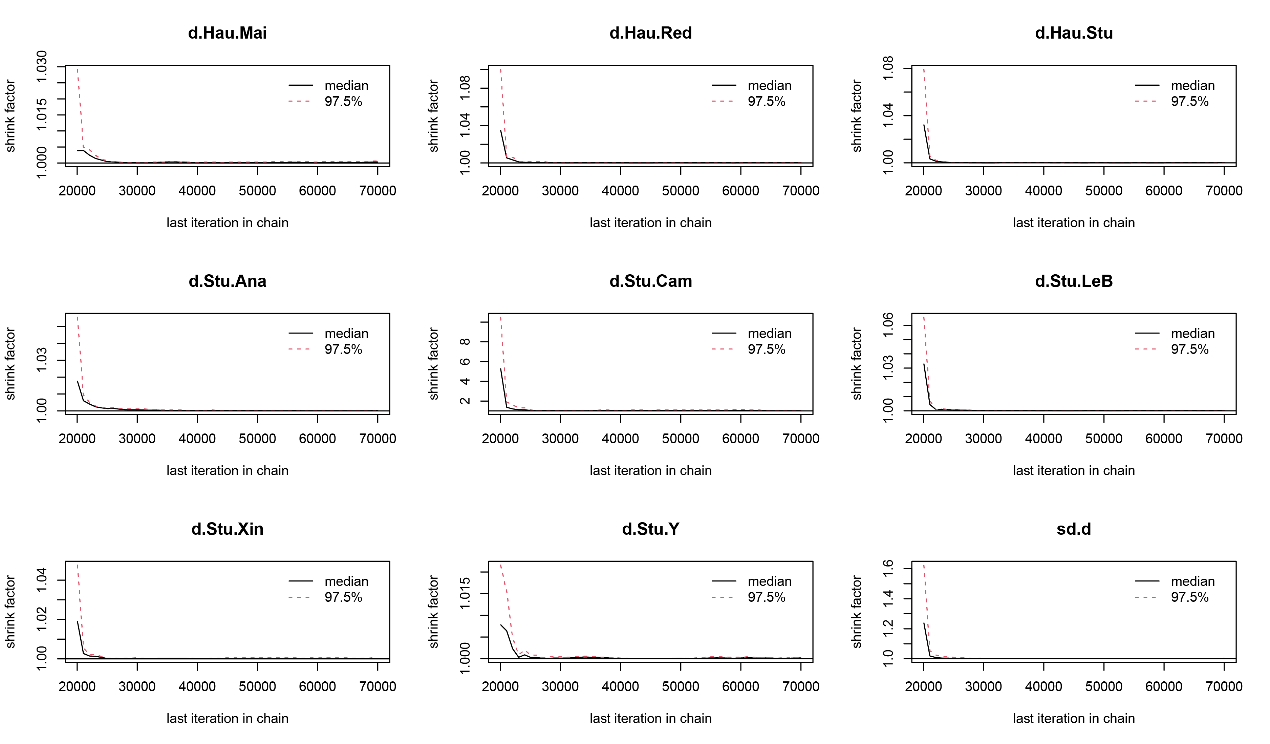


**Supplementary Figure S18**
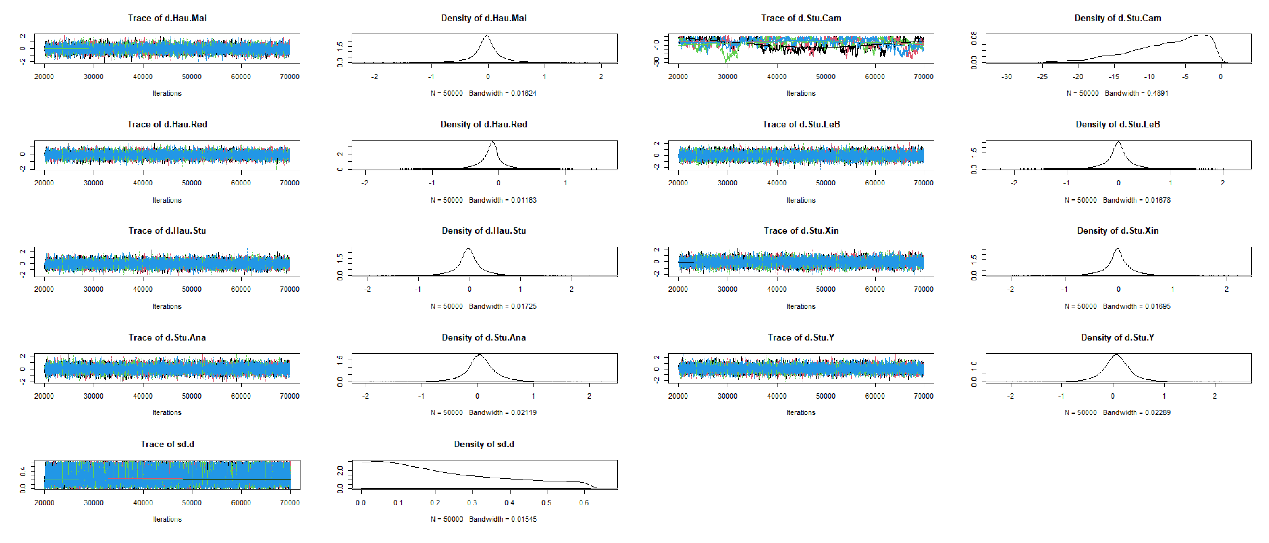


**Supplementary Figure S19**
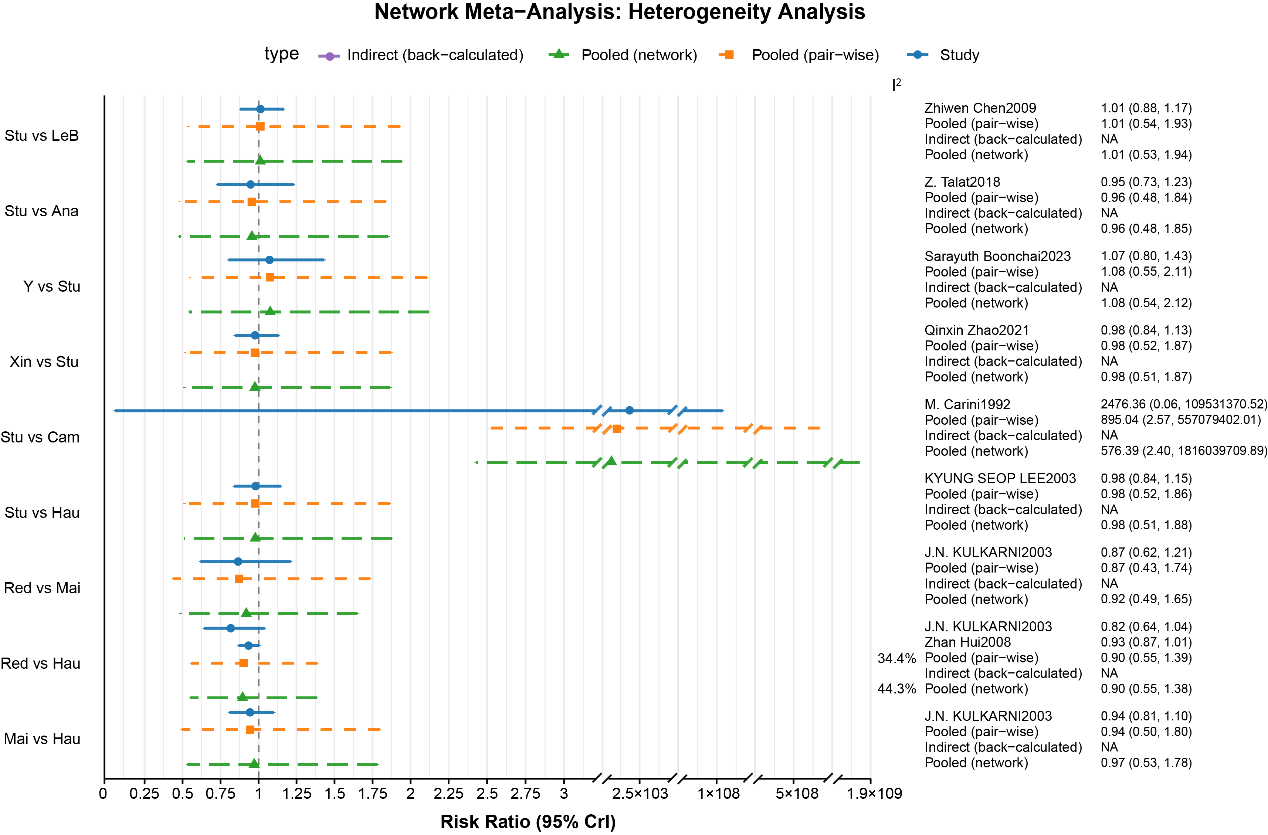


**Supplementary Figure S20**
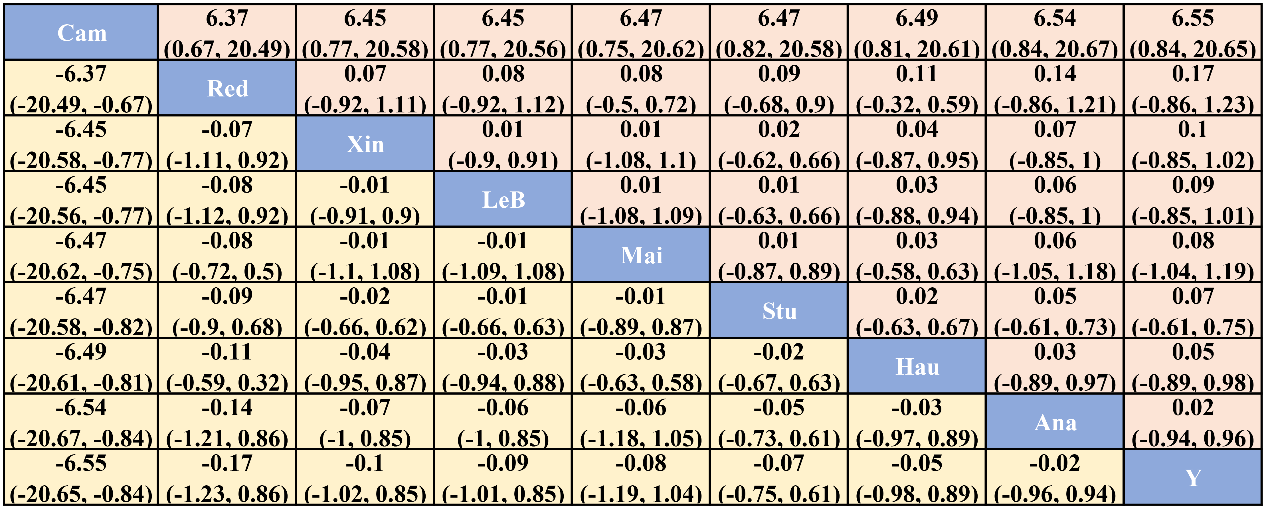


**Supplementary Figure S21**
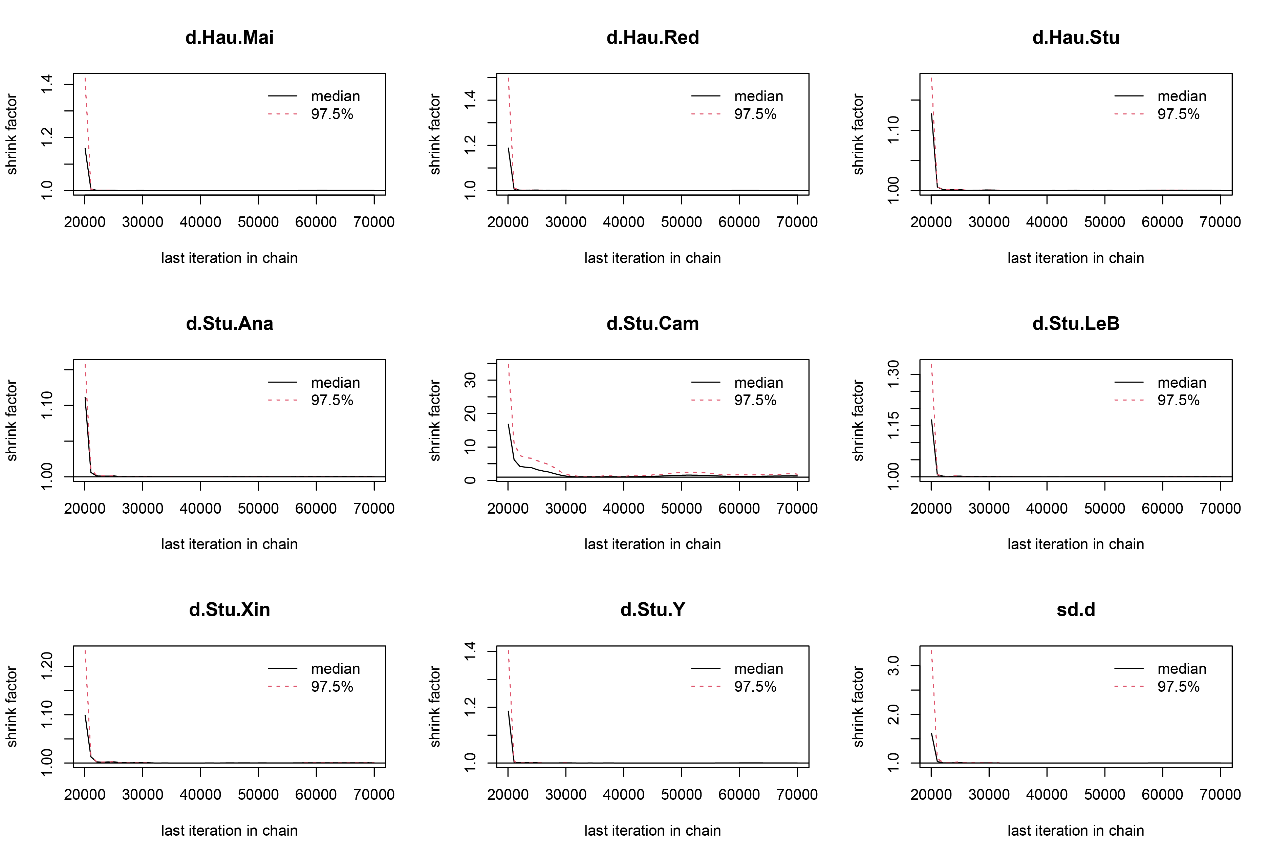


**Supplementary Figure S22**
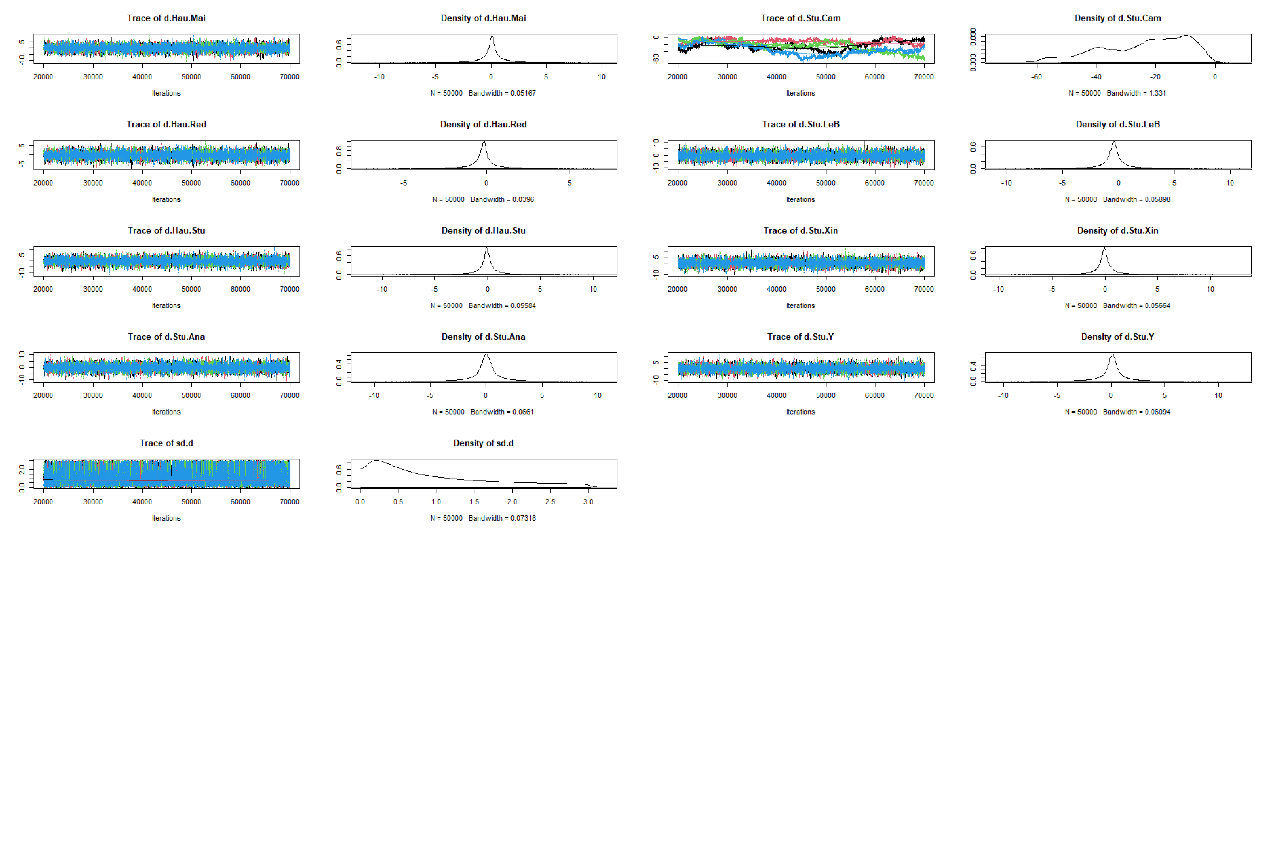


**Supplementary Figure S23**


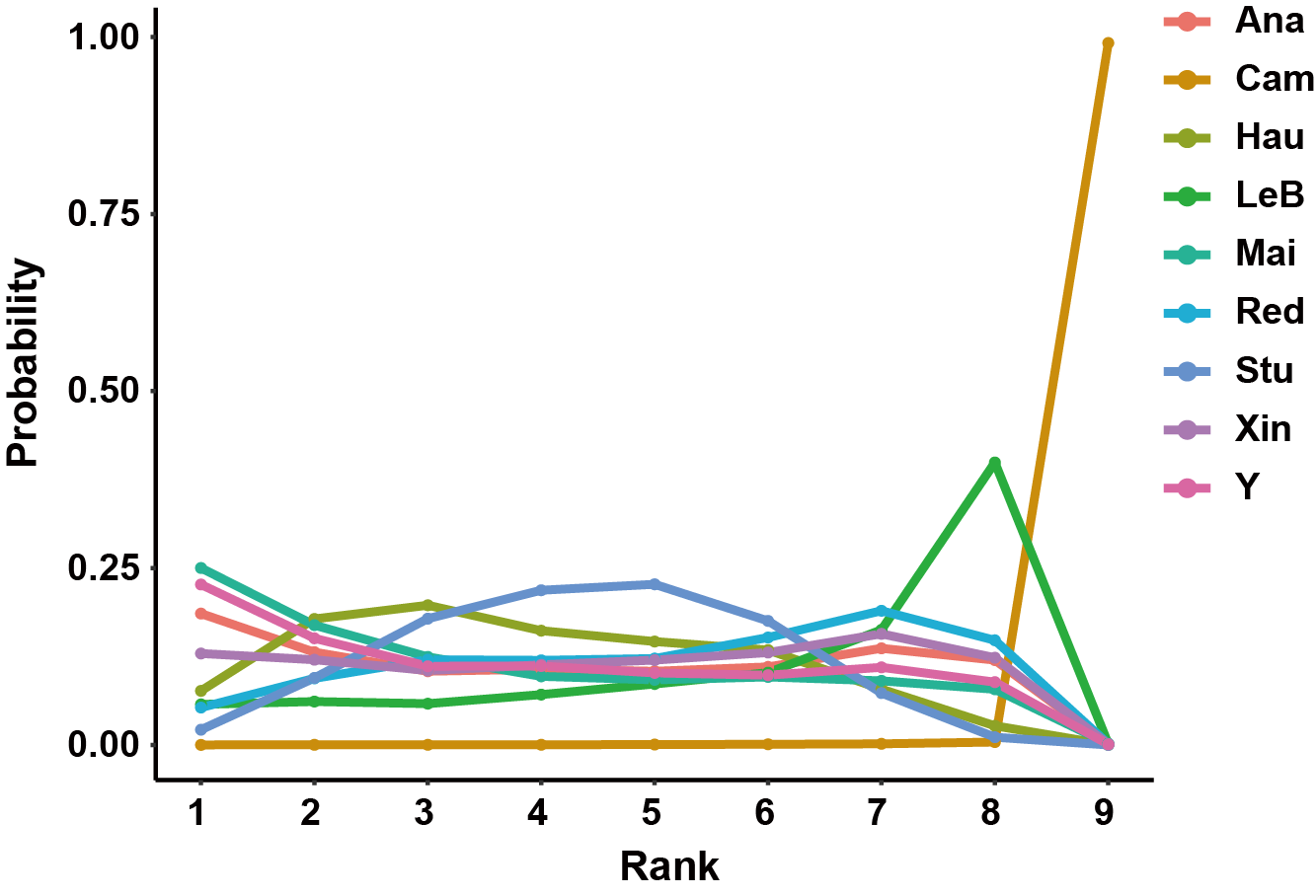


**Supplementary Figure S24**


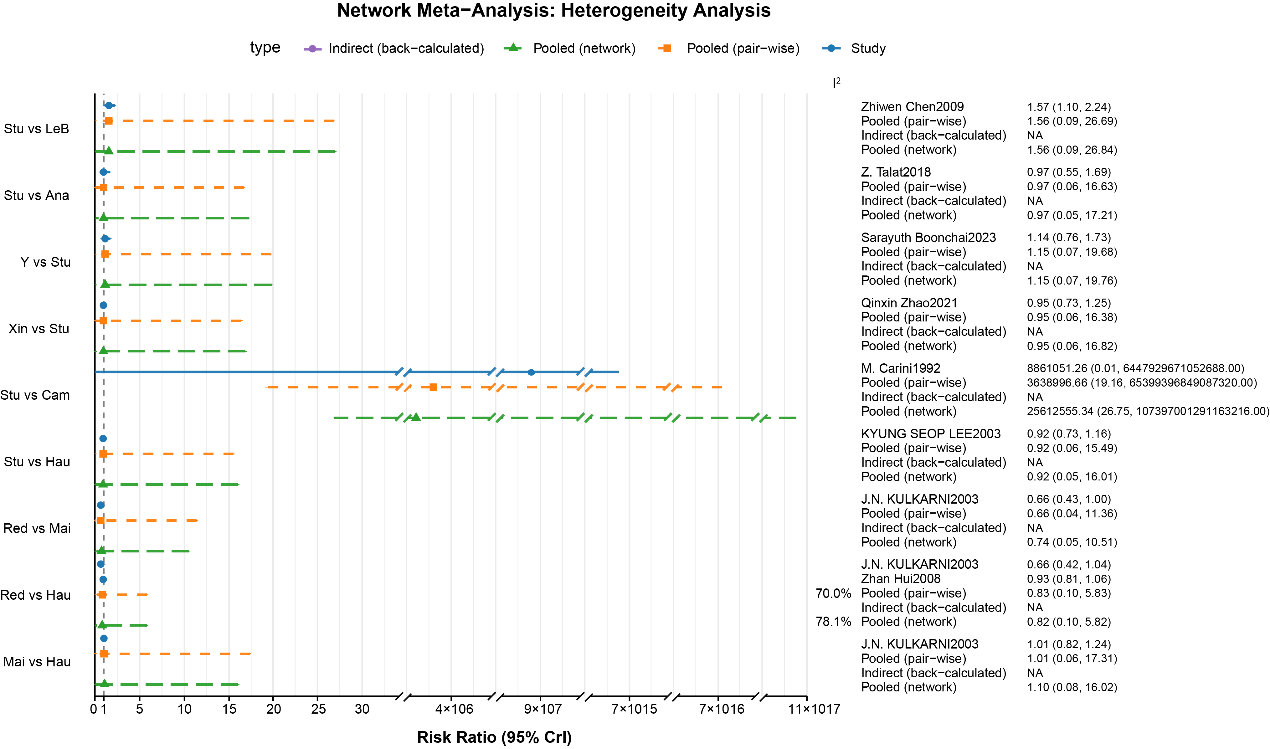


**Supplementary Figure S25**
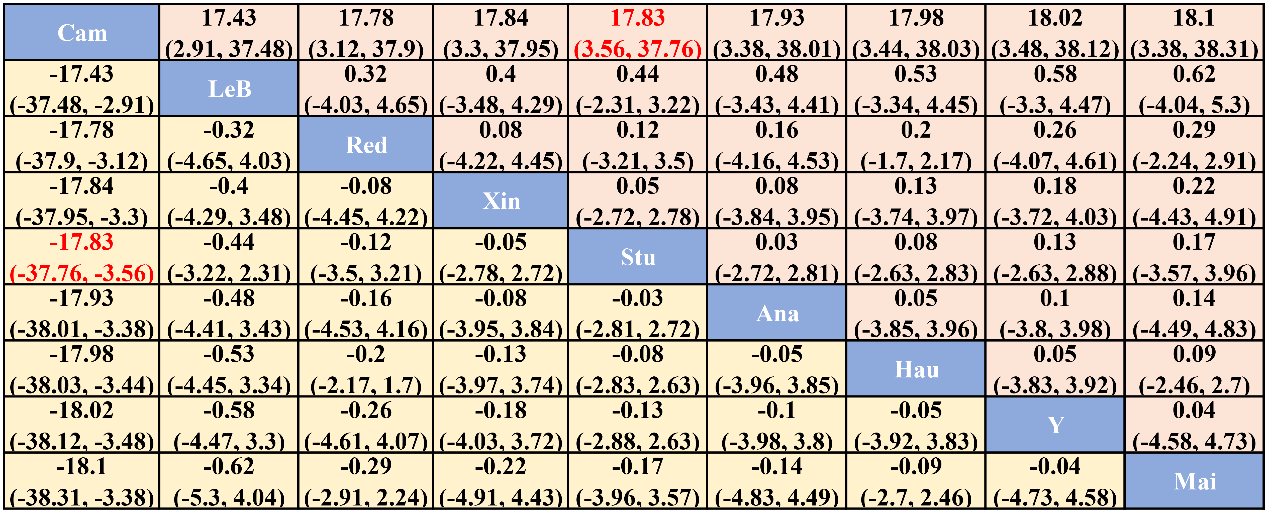


**Supplementary Figure Legends**

**Supplementary Figure S1. Risk of bias assessment for included studies**

(A) Risk of bias assessment of the three randomized controlled trials (RCTs) using the Cochrane Collaboration’s Risk of Bias tool across 7 standard domains. (B) Summary of risk of bias judgments for the RCTs, presented as the proportion of studies rated as low, unclear, or high risk for each domain. (C) Methodological quality assessment of the included cohort studies based on the Newcastle–Ottawa Scale (NOS), with stars indicating study quality across selection, comparability, and outcome domains.

**Supplementary Figure S2.** **Gelman-Rubin diagnostic plots of maximum neobladder capacity (MNC).**

Convergence diagnostics for the Markov chain Monte Carlo (MCMC) simulations of pairwise comparisons for MNC are shown, with the Studer pouch used as the reference treatment.

**Abbreviations:** Hau, Hautmann pouch; Koc, Kock pouch; Ile, Ileocecal pouch; Ind, Indiana pouch; Stu, Studer pouch; LeB, Le Bag pouch; Red, Reddy pouch; Cam, Camey I pouch; Gol, Goldwasser pouch; T, T pouch; Y, Y pouch; sd.d, between-study standard deviation; d, effect difference.

**Supplementary Figure S3. Trace and density plots for maximum neobladder capacity (MNC).**

The Markov chain Monte Carlo (MCMC) trace plots (left panels) and corresponding posterior density distributions (right panels) for relative treatment effects on MNC, with the Studer pouch as the reference, along with the between-study heterogeneity parameter.

**Abbreviations:** Hau, Hautmann pouch; Koc, Kock pouch; Ile, Ileocecal pouch; Ind, Indiana pouch; Stu, Studer pouch; LeB, Le Bag pouch; Red, Reddy pouch; Cam, Camey I pouch; Gol, Goldwasser pouch; T, T pouch; Y, Y pouch; sd.d, between-study standard deviation; d, effect difference; N, number of iterations.

**Supplementary Figure S4. Ranking probably plot of maximum neobladder capacity (MNC).**

The figure illustrates the rank probability distributions of the included orthotopic neobladder reconstruction techniques for MNC, derived from the surface under the cumulative ranking curve (SUCRA) in the Bayesian network meta-analysis. For each technique, the probability of achieving each possible rank is displayed.

**Abbreviations:** Cam, Camey I pouch; Gol, Goldwasser pouch; Hau, Hautmann pouch; Ile, Ileocecal pouch; Ind, Indiana pouch; Koc, Kock pouch; LeB, Le Bag pouch; Red, Reddy pouch; Stu, Studer pouch; T, T pouch; Y, Y pouch.

**Supplementary Figure S5. Heterogeneity analysis for maximum neobladder capacity (MNC).**

This figure presents the heterogeneity assessment of pairwise comparisons for MNC within the network meta-analysis. It displays individual study effect together with pooled pairwise estimates, indirect back-calculated, and pooled network estimates, expressed as mean differences (MDs) with 95% credible intervals (CrIs). The vertical dashed line denotes the line of no effect. Estimates are derived from a random-effects Bayesian model. Between-study heterogeneity for each comparison is quantified using the I² statistic.

**Abbreviations:** Stu, Studer pouch; LeB, Le Bag pouch; Koc, Kock pouch; Hau, Hautmann pouch; Y, Y pouch; Ile, Ileocecal pouch; Cam, Camey I pouch; T, T pouch; Gol, Goldwasser pouch; Ind, Indiana pouch; Red, Reddy pouch; CrI, credible interval.

**Supplementary Figure S6. League table of pairwise comparisons for maximum neobladder capacity (MNC).**

The table displays the relative treatment effects among orthotopic neobladder reconstruction techniques for the core functional outcome of MNC, expressed as mean differences (MDs) with 95% credible intervals (CrIs). Each cell represents the estimated difference in MNC between the technique listed in the row and that in the column. Positive MD values favor the column-defining technique, whereas negative values favor the row-defining technique. Cells highlighted in red indicate a statistically significant difference in NC between the two techniques. Estimates were obtained from a random-effects Bayesian network meta-analysis.

**Abbreviations:** Ind, Indiana pouch; Ile, Ileocecal pouch; Red, Reddy pouch; Cam, Camey I pouch; Y, Y pouch; Stu, Studer pouch; LeB, Le Bag pouch; Koc, Kock pouch; Hau, Hautmann pouch; Gol, Goldwasser pouch; T, T pouch.

**Supplementary Figure S7. Gelman-Rubin diagnostic plots of maximal flow rate** **(MFR).**

Convergence diagnostics for the Markov chain Monte Carlo (MCMC) simulations of pairwise comparisons for MFR are shown, with the Studer pouch used as the reference treatment.

**Abbreviations:** Hau, Hautmann pouch; Stu, Studer pouch; LeB, Le Bag pouch; Red, Reddy pouch; Gol, Goldwasser pouch; Xin, Xing pouch; Mai, Mainz pouch; sd.d, between-study standard deviation; d, effect difference.

**Supplementary Figure S8. Trace and density plots of maximal flow rate** **(MFR).**

The Markov chain Monte Carlo (MCMC) trace plots (left panels) and corresponding posterior density distributions (right panels) for relative treatment effects on MFR, with the Studer pouch as the reference, along with the between-study heterogeneity parameter.

**Abbreviations:** Hau, Hautmann pouch; Stu, Studer pouch; LeB, Le Bag pouch; Red, Reddy pouch; Gol, Goldwasser pouch; Xin, Xing pouch; Mai, Mainz pouch; sd.d, between-study standard deviation; d, effect difference; N, number of iterations.

**Supplementary Figure S9. Ranking probably plot of maximal flow rate** **(MFR).**

The figure illustrates the rank probability distributions of the included orthotopic neobladder reconstruction techniques for MFR, derived from the surface under the cumulative ranking curve (SUCRA) in the Bayesian network meta-analysis. For each technique, the probability of achieving each possible rank is displayed.

**Abbreviations:** Hau, Hautmann pouch; Stu, Studer pouch; LeB, Le Bag pouch; Red, Reddy pouch; Gol, Goldwasser pouch; Xin, Xing pouch; Mai, Mainz pouch.

**Supplementary Figure S10. Heterogeneity analysis for maximal flow rate** **(MFR).**

This figure presents the heterogeneity assessment of pairwise comparisons for MFR within the network meta-analysis. It displays individual study effect together with pooled pairwise estimates, indirect back-calculated, and pooled network estimates, expressed as mean differences (MDs) with 95% credible intervals (CrIs). The vertical dashed line denotes the line of no effect. Estimates are derived from a random-effects Bayesian model. Between-study heterogeneity for each comparison is quantified using the I² statistic.

**Abbreviations:** Hau, Hautmann pouch; Stu, Studer pouch; LeB, Le Bag pouch; Red, Reddy pouch; Gol, Goldwasser pouch; Xin, Xing pouch; Mai, Mainz pouch; CrI, credible interval.

**Supplementary Figure S11. League table of pairwise comparisons for maximal flow rate** **(MFR).**

The table displays the relative treatment effects among orthotopic neobladder reconstruction techniques for the core functional outcome of MFR, expressed as mean differences (MDs) with 95% credible intervals (CrIs). Each cell represents the estimated difference in MFR between the technique listed in the row and that in the column. Positive MD values favor the column-defining technique, whereas negative values favor the row-defining technique. Cells highlighted in red indicate a statistically significant difference in NC between the two techniques. Estimates were obtained from a random-effects Bayesian network meta-analysis.

**Abbreviations:** Hau, Hautmann pouch; Stu, Studer pouch; LeB, Le Bag pouch; Red, Reddy pouch; Gol, Goldwasser pouch; Xin, Xing pouch; Mai, Mainz pouch.

**Supplementary Figure S12. Gelman-Rubin diagnostic plots of postvoid residual volume (PVR).**

Convergence diagnostics for the Markov chain Monte Carlo (MCMC) simulations of pairwise comparisons for PVR are shown, with the Studer pouch used as the reference treatment.

**Abbreviations:** Hau, Hautmann pouch; Stu, Studer pouch; LeB, Le Bag pouch; Red, Reddy pouch; Gol, Goldwasser pouch; Y, Y pouch; Ile, Ileocecal pouch; Mai, Mainz pouch; sd.d, between-study standard deviation; d, effect difference.

**Supplementary Figure S13. Trace and density plots of postvoid residual volume (PVR).**

The Markov chain Monte Carlo (MCMC) trace plots (left panels) and corresponding posterior density distributions (right panels) for relative treatment effects on PVR, with the Studer pouch as the reference, along with the between-study heterogeneity parameter.

**Abbreviations:** Hau, Hautmann pouch; Stu, Studer pouch; LeB, Le Bag pouch; Red, Reddy pouch; Gol, Goldwasser pouch; Y, Y pouch; Ile, Ileocecal pouch; Mai, Mainz pouch; sd.d, between-study standard deviation; d, effect difference; N, number of iterations.

**Supplementary Figure S14. Ranking probably plot of postvoid residual volume (PVR).**

The figure illustrates the rank probability distributions of the included orthotopic neobladder reconstruction techniques for PVR, derived from the surface under the cumulative ranking curve (SUCRA) in the Bayesian network meta-analysis. For each technique, the probability of achieving each possible rank is displayed.

**Abbreviations:** Hau, Hautmann pouch; Stu, Studer pouch; LeB, Le Bag pouch; Red, Reddy pouch; Gol, Goldwasser pouch; Y, Y pouch; Ile, Ileocecal pouch; Mai, Mainz pouch.

**Supplementary Figure S15. Heterogeneity analysis for postvoid residual volume (PVR).**

This figure presents the heterogeneity assessment of pairwise comparisons for PVR within the network meta-analysis. It displays individual study effect together with pooled pairwise estimates, indirect back-calculated, and pooled network estimates, expressed as mean differences (MDs) with 95% credible intervals (CrIs). The vertical dashed line denotes the line of no effect. Estimates are derived from a random-effects Bayesian model. Between-study heterogeneity for each comparison is quantified using the I² statistic.

**Abbreviations:** Hau, Hautmann pouch; Stu, Studer pouch; LeB, Le Bag pouch; Red, Reddy pouch; Gol, Goldwasser pouch; Y, Y pouch; Ile, Ileocecal pouch; Mai, Mainz pouch; CrI, credible interval.

**Supplementary Figure S16. League table of pairwise comparisons for postvoid residual volume (PVR).**

The table displays the relative treatment effects among orthotopic neobladder reconstruction techniques for the core functional outcome of PVR, expressed as mean differences (MDs) with 95% credible intervals (CrIs). Each cell represents the estimated difference in PVR between the technique listed in the row and that in the column. Positive MD values favor the column-defining technique, whereas negative values favor the row-defining technique. Cells highlighted in red indicate a statistically significant difference in NC between the two techniques. Estimates were obtained from a random-effects Bayesian network meta-analysis.

**Abbreviations:** Hau, Hautmann pouch; Stu, Studer pouch; LeB, Le Bag pouch; Red, Reddy pouch; Gol, Goldwasser pouch; Y, Y pouch; Ile, Ileocecal pouch; Mai, Mainz pouch.

**Supplementary Figure S17. Gelman-Rubin diagnostic plots of daytime continence (DC).**

Convergence diagnostics for the Markov chain Monte Carlo (MCMC) simulations of pairwise comparisons for DC are shown, with the Studer pouch used as the reference treatment.

**Abbreviations:** Hau, Hautmann pouch; Mai, Mainz pouch; Red, Reddy pouch; Stu, Studer pouch; Ana, Anatolian pouch; Cam, Camey I pouch; LeB, Le Bag pouch; Xin, Xing pouch; Y, Y pouch; sd.d, between-study standard deviation; d, effect difference.

**Supplementary Figure S18. Trace and density plots of daytime continence (DC).**

The Markov chain Monte Carlo (MCMC) trace plots (left panels) and corresponding posterior density distributions (right panels) for relative treatment effects on DC, with the Studer pouch as the reference, along with the between-study heterogeneity parameter.

**Abbreviations:** Hau, Hautmann pouch; Mai, Mainz pouch; Red, Reddy pouch; Stu, Studer pouch; Ana, Anatolian pouch; Cam, Camey I pouch; LeB, Le Bag pouch; Xin, Xing pouch; Y, Y pouch; sd.d, between-study standard deviation; d, effect difference; N, number of iterations.

**Supplementary Figure S19. Heterogeneity analysis for daytime continence (DC).**

This figure presents the heterogeneity assessment of pairwise comparisons for DC within the network meta-analysis. It displays individual study effect together with pooled pairwise estimates, indirect back-calculated, and pooled network estimates, expressed as risk ratios (RRs) with 95% credible intervals (CrIs). The vertical dashed line denotes the line of no effect. Estimates are derived from a random-effects Bayesian model. Between-study heterogeneity for each comparison is quantified using the I² statistic.

**Abbreviations:** Stu, Studer pouch; LeB, Le Bag pouch; Ana, Anatolian pouch; Y, Y pouch; Xin, Xing pouch; Cam, Camey I pouch; Hau, Hautmann pouch; Red, Reddy pouch; Mai, Mainz pouch; sd.d, between-study standard deviation; d, effect difference; CrI, credible interval.

**Supplementary Figure S20. League table of pairwise comparisons for daytime continence (DC).**

The table displays the relative treatment effects among orthotopic neobladder reconstruction techniques for the core functional outcome of DC, expressed as risk ratios (RRs) with 95% credible intervals (CrIs). Each cell represents the estimated difference in DC between the technique listed in the row and that in the column. Positive values favor the column-defining technique, whereas negative values favor the row-defining technique. Cells highlighted in red indicate a statistically significant difference in NC between the two techniques. Estimates were obtained from a random-effects Bayesian network meta-analysis.

**Abbreviations:** Cam, Camey I pouch; Red, Reddy pouch; Xin, Xing pouch; LeB, Le Bag pouch; Mai, Mainz pouch; Stu, Studer pouch; Hau, Hautmann pouch; Ana, Anatolian pouch; Y, Y pouch.

**Supplementary Figure S21. Gelman-Rubin diagnostic plots of nighttime continence (NC).**

Convergence diagnostics for the Markov chain Monte Carlo (MCMC) simulations of pairwise comparisons for NC are shown, with the Studer pouch used as the reference treatment.

**Abbreviations:** Hau, Hautmann pouch; Mai, Mainz pouch; Red, Reddy pouch; Stu, Studer pouch; Ana, Anatolian pouch; Cam, Camey I pouch; LeB, Le Bag pouch; Xin, Xing pouch; Y, Y pouch; sd.d, between-study standard deviation; d, effect difference.

**Supplementary Figure S22. Trace and density plots of nighttime continence (NC).**

The Markov chain Monte Carlo (MCMC) trace plots (left panels) and corresponding posterior density distributions (right panels) for relative treatment effects on NC, with the Studer pouch as the reference, along with the between-study heterogeneity parameter.

**Abbreviations:** Hau, Hautmann pouch; Mai, Mainz pouch; Red, Reddy pouch; Stu, Studer pouch; Ana, Anatolian pouch; Cam, Camey I pouch; LeB, Le Bag pouch; Xin, Xing pouch; Y, Y pouch; sd.d, between-study standard deviation; d, effect difference; N, number of iterations.

**Supplementary Figure S23. Ranking probably plot of nighttime continence (NC).**

The figure illustrates the rank probability distributions of the included orthotopic neobladder reconstruction techniques for NC, derived from the surface under the cumulative ranking curve (SUCRA) in the Bayesian network meta-analysis. For each technique, the probability of achieving each possible rank is displayed.

**Abbreviations:** Ana, Anatolian pouch; Cam, Camey I pouch; Hau, Hautmann pouch; LeB, Le Bag pouch; Mai, Mainz pouch; Red, Reddy pouch; Stu, Studer pouch; Xin, Xing pouch; Y, Y pouch.

**Supplementary Figure S24. Heterogeneity analysis for nighttime continence (NC).**

This figure presents the heterogeneity assessment of pairwise comparisons for DC within the network meta-analysis. It displays individual study effect together with pooled pairwise estimates, indirect back-calculated, and pooled network estimates, expressed as risk ratios (RRs) with 95% credible intervals (CrIs). The vertical dashed line denotes the line of no effect. Estimates are derived from a random-effects Bayesian model. Between-study heterogeneity for each comparison is quantified using the I² statistic.

**Abbreviations:** Stu, Studer pouch; LeB, Le Bag pouch; Ana, Anatolian pouch; Y, Y pouch; Xin, Xing pouch; Cam, Camey I pouch; Hau, Hautmann pouch; Red, Reddy pouch; Mai, Mainz pouch; sd.d, between-study standard deviation; d, effect difference; CrI, credible interval.

**Supplementary Figure S25. League table of pairwise comparisons for nighttime continence (NC).**

The table displays the relative treatment effects among orthotopic neobladder reconstruction techniques for the core functional outcome of NC, expressed as risk ratios (RRs) with 95% credible intervals (CrIs). Each cell represents the estimated difference in NC between the technique listed in the row and that in the column. Positive values favor the column-defining technique, whereas negative values favor the row-defining technique. Cells highlighted in red indicate a statistically significant difference in NC between the two techniques. Estimates were obtained from a random-effects Bayesian network meta-analysis.

**Abbreviations:** Cam, Camey I pouch; LeB, Le Bag pouch; Red, Reddy pouch; Xin, Xing pouch; Stu, Studer pouch; Ana, Anatolian pouch; Hau, Hautmann pouch; Y, Y pouch; Mai, Mainz pouch.
